# Mechanochemistry to Form Substituted Imidazoles, Imidazolium Salts and NHC–Gold Complexes with Fluorine-Containing Groups

**DOI:** 10.3390/molecules30030522

**Published:** 2025-01-24

**Authors:** Chloé Salis, Sabrina Mohammedi, Lucia Turazza, Yuna Blandin, Maritie Garnier, Catherine Hemmert, Michel Baltas, Heinz Gornitzka

**Affiliations:** LCC-CNRS, Université de Toulouse, CNRS, UPS, 31077 Toulouse, France; salis@lcc-toulouse.fr (C.S.); sabrinamohammedi@outlook.com (S.M.); lucia-turazza.turazza@univ-tlse3.fr (L.T.); yuna.blandin@univ-tlse3.fr (Y.B.); maritie.garnier@lcc-toulouse.fr (M.G.); hemmert@lcc-toulouse.fr (C.H.)

**Keywords:** mechanochemistry, gold, imidazole, imidazolium salts, *N*-heterocyclic carbenes

## Abstract

Synthesis of organometallic compounds has had an enormous impact on medicine. In this context, gold complexes are gaining much interest since the discovery of the cytotoxic effect of cisplatin. On the other hand, in the last two decades, the mechanochemical synthetic approaches have been developed considerably demonstrating that they could also be a powerful tool enabling environmentally benign and sustainable synthesis of metal complexes. The present work focuses on mechanochemical synthesis of precursors and gold–NHC complexes of type NHCAuCl and [AubisNHC]^+^. The mechanochemical approach has been studied to afford four substituted imidazoles, eight imidazolium salts and six NHCAuCl and one [AubisNHC]^+^. Substituted imidazoles were obtained with yields varying between 29–99%. Five imidazolium salts bearing aliphatic carbon atoms were obtained, with yields from 46–81%. It is important to notice that the reaction can follow the aging process giving rise to imidazolium salts in very good yields. Concerning the gold(I) complexes, for the first time, six mono NHC complexes of type NHCAuCl have been synthesized, five of them with yields varying between 41–83%. Finally, compound **19** [AubisNHC]^+^ has been obtained not only by transmetallation, but most gratifyingly through direct metalation in 73% yield.

## 1. Introduction

For two decades, new approaches in the area of organic and organometallic synthesis oriented towards biologically active compounds have integrated the need for more efficient and environmentally safe methods. In fact, since 2000, many regulations for the chemical and pharmaceutical industries have appeared, especially in terms of efficiency, waste management, and energy input. All these issues are termed “Green Chemistry”, a multifaceted field dealing with what we call the twelve principles of Anastas and Warner [1]. Conventional reactions procedures are classically conducted in solutions (mostly organic), usually under reflux. The Green Chemistry approaches focus on the use of alternative energy sources and their efficiency, these sources being essential: photochemistry through light excitation, microwave, sonochemistry irradiation, and mechanochemistry [2].

Mechanochemistry has rapidly emerged as a powerful tool enabling environmentally benign and sustainable synthesis of metal complexes across the periodic table. In 2023, Aleksanyan and Kozlov reported the opportunities and prospects of different mechanochemical tools in the synthesis of organometallic compounds, including transition metal complexes with *N*-heterocyclic carbene, arene, and cyclopentadienyl ligands, mono-metalacyclic and pincer derivatives, as well as main group metal compounds (e.g., allyl complexes and the Grignard reagents) [3]. Many important organometallic transformations such as C–H bond metalation, trans-metalation, and oxidative addition can be successfully implemented in a highly productive and energy-efficient way.

In pharmaceutical chemistry, metal complexes are gaining more and more interest since the discovery of the cytotoxic effect of cisplatin [4,5]. In this context, the number of publications concerning gold compounds for biomedical applications increased significantly the last 10 years. Different ligands can be used to stabilize and to protect the gold cation under physiological conditions. The most applied are phosphines, thiocarbamates, and *N*-heterocyclic carbenes (NHCs) [6,7,8,9,10,11].

Particularly, in the field of the application of mechanochemistry to the synthesis of NHCs complexes, we can mention the pioneering work of Bantreil, Métro, Lamaty et al. concerning the efficient synthesis of *N*,*N*-dialkylimidazolium halides under solvent-free mechanochemical conditions [12]. These precursors, obtained in short reaction times and in yields of 79–99%, were then subjected to react with silver(I) oxide, thus providing silver(I) carbene complexes in good to excellent yields. Furthermore, for one example, the authors proceeded efficiently in a one-pot three-step ball-milling transmetalation reaction with Au(SMe_2_)Cl affording the corresponding gold(I) complex.

Two years later, the same group reported the solvent-free mechanosynthetic strategy of *N*,*N*-diaryl NHCs and their transmetalation to gold, copper, or palladium complexes. The gold complex was obtained in a one-pot two-step procedure in 76% yield [13].

In 2020, Pisanò and Cazin reported the synthesis of NHCCuCl complexes by direct metalation using K_2_CO_3_ as base [14]. One year later, the same authors published a general mechanochemical protocol for the synthesis of late-transition metal mono-NHC complexes [15].

In 2022, Tubaro and co-workers have shown that mechanochemistry can also be used to make ligand exchange reactions on dinuclear gold complexes [16].

For a couple of years, our team has worked on gold–NHC complexes for biomedical applications, neutral complexes of type NHCAuCl for leishmania [17], and cationic [AubisNHC]^+^ complexes for cancer [18,19]. We found highly interesting results on leishmania for neutral complexes containing fluorinated aromatic substituents on the NHC ligands [20]. The main problem regarding the synthesis of such complexes is that the reaction conditions for each step sometimes demand a couple of days and high temperatures. In order to possibly circumvent these difficulties and with respect to Green Chemistry approaches, we wish to report here our findings when applying solvent-free ball-milling conditions for each step of our synthetic route. Our study concerns synthesis of substituted imidazoles starting from imidazole, formation of imidazolium salts, and transformation of imidazolium salts into NHC ligands coordinating gold(I) centers.

## 2. Results and Discussion

### 2.1. Synthesis of Substituted Imidazoles ***1*** to ***4***

In a conventional way, the reaction of imidazole with halogenated aliphatic groups is performed in the presence of bases (NaOH or KOH) in polar solvents as THF, at high temperatures with reaction times between 24 h and 120 h. For halogenated aromatic systems, even more drastic conditions are needed by melting the starting reagents at 205 °C in the presence of K_2_CO_3_ and CuSO_4_. The yields of the substituted imidazoles obtained after silica gel purification can increase to 99%.

**Scheme 1:**
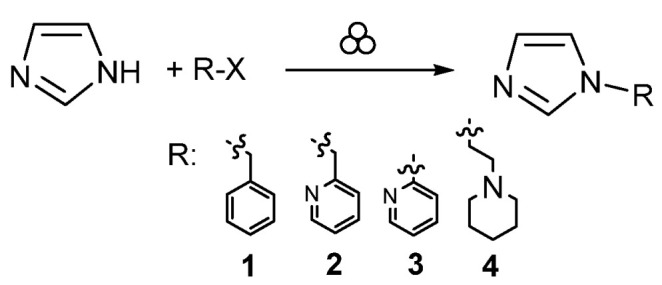
Synthesis of substituted imidazoles **1** to **4**.

In this work, we have first undertaken the mechanochemical study concerning the synthesis for the first time of four substituted imidazoles (Scheme 1).

The coupling between benzyl chloride and imidazole that already affords very good results by classical synthesis (yield 95%) [21] was first examined. For this reaction, reagents were ground in a Planetary Mill Pulverisette 7 (P7, Fritsch) equipped with two 20 mL stainless steel bowls, each bowl equipped with 5 balls of 10 mm diameter. Grinding times, rounds per minute, stoichiometries, and presence of different bases have been varied. Conditions and yields are summarized in Table 1. While a 1:1 eq of imidazole and benzyl chloride afforded a sluggish result (46% of conversion and 18% yield observed, entry 1, Table 1), when using at least one more equivalent of imidazole as reagent and base for trapping HCl formed, we obtained better (Table 1, entries 2–4) results. Similar conversion was also determined when operating in the presence of 5:1 eq of imidazole/benzyl chloride, the same duration as before but by applying less energy (500 rpm instead of 800 rpm, Table 1, entry 5). The yields obtained with the excess of imidazole after silica gel purification ranged from 39–99%. When using 1 eq of imidazole and adding 1 eq of an inorganic base, we found also similar or better results. In fact, when reacting imidazole/benzyl chloride/K_2_CO_3_ in a 1/1/1 ratio for 4 × 45 (5′) at 800 rpm, a 43% yield was obtained (Table 1, entry 6). NaOH (1.6 eq) or KOH (2 eq) yielded the best results, the highest yield after silica gel purification being obtained using 2 eq of KOH (76%, Table 1, entry 8).

We next focused on the reaction in order to obtain substituted imidazoles **2**–**4**. The halogenated reagents include 2-(bromomethyl)pyridine hydrobromide, 2-bromopyridine, and 1-(2-chloroethyl)piperidine hydrochloride (for synthesis of compounds **2**, **3,** and **4**, respectively). The main conditions found previously, i.e., duration four times 45 min with 5 min pause and at 800 rpm, were applied.

When grinding imidazole with 2-(bromomethyl)pyridine hydrobromide in a 5:1 ratio, the best conditions found for **1**, the conversion observed is very low (14% estimated by ^1^H NMR) and no pure product could be obtained (Table 2, entry 9). The reaction of a 1:1 eq of reagents in the presence of 2 eq of KOH afforded the desired substituted imidazole **2** in 55% yield after silica gel purification (Table 2, entry 10). Under these conditions and as reagents are solids, we experimented a Liquid Assisted Grinding (LAG) procedure by using THF which is a usual solvent for these reactions in solution. The LAG experiments under three different reagents and reagents/base ratio conditions led to much better results with yields for compound **2** after silica gel purification varying between 68–72% (Table 2, entries 11, 12, and 13).

When grinding imidazole with 2-bromopyridine in a 1:1 ratio, no reaction was observed in the absence or presence of base (KOH or K_2_CO_3_) and also in one case in the presence of copper sulfate (0.1 eq) aimed to promote the SNAr reaction (Table 2, entries 14–16). Gratifyingly, when using a 5:1 ratio of the two reagents and copper powder (0.2 eq), the desired substituted imidazole **3** was obtained with a yield of 50%. We obtained similar results when operating in the presence of KOH or under LAG (THF) conditions with 800 rpm or 25 Hz (Table 2, entries 17–19).

When grinding imidazole with 1-(2-chloroethyl) piperidine hydrochloride in a 5:1 ratio (Table 2, entry 20), the desired compound **4** is obtained in 64% yield. When adding 1 eq of KOH, the yield is the same (64%, Table 2, entry 21).

### 2.2. Synthesis of Imidazolium Salts

**Scheme 2:**
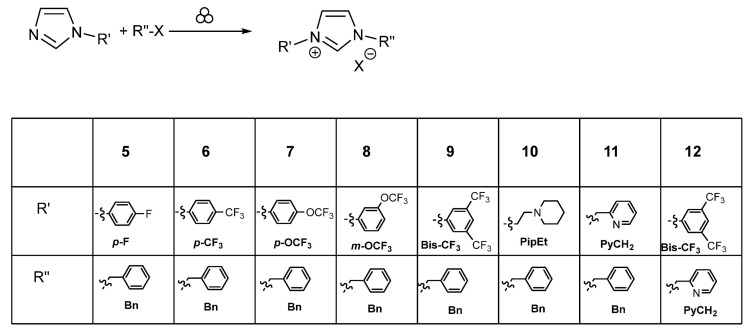
Synthesis of imidazolium salts **5** to **12**. The grinding conditions are mentioned in Table 3.

We next wished to evaluate the efficiency of the grinding approach in order to obtain imidazolium salts when reacting benzylchloride or 2-(bromomethyl)pyridine hydrobromide with substituted imidazoles bearing fluorinated aromatic systems on the nitrogen atom of imidazole (Scheme 2). The latter compounds were synthesized according to the protocols reported by us [20].

Concerning the synthesis of these derivatives, we operated in two different mills: the Mixer Mill MM400 (Retsch, Haan, Germany) equipped with two 10 mL stainless steel jars, each jar with 2 balls of 10 mm Ø and at max working frequency of 10 Hz or 25 Hz, and the Planetary Mill Pulverisette 7 (P7) (Fritsch, Fellbach, Germany). All reactions have been conducted (either manually or by programming) with four cycles of 45 min ball milling with interruptions of 5 min (4 × 45 (5′)).

If not otherwise mentioned, the reactions have been conducted with a ratio of 1:1.1 eq between the imidazole and the halogenated reagent. Conversions have been estimated by ^1^H-NMR spectroscopy based on the integration of the CH_2_-groups of the benzyl or the PyCH_2_ of the halogenated reagents and the formed imidazolium salts.

The Planetary Mill Pulverisette 7 (P7) at 800 rpm has been used to react p-fluorophenyl imidazole, p-trifluoromethylphenyl imidazole, and p-trifluoromethoxy phenyl imidazole with benzyl chloride, leading to the corresponding imidazolium salts **5** to **7** with conversions of 42%, 69%, and 46% and yields of 18%, 60%, and 46%, respectively (entries 22, 23, and 25). For m-trifluoromethoxy phenyl imidazole reacting with benzyl chloride, better results were obtained with the Mixer Mill MM400 at 25 Hz frequency (conversion 42%, yield 41%) to yield compound **8** (entry 26). By applying a lower frequency (10 Hz), the conversion (7%) and yield (13%) drop considerably (entry 27). Under the same conditions (Mixer Mill MM400 at 25 Hz frequency), 3,5-bis-trifluorophenyl imidazole does not react with benzyl chloride (entry 28); the same could be observed using the Planetary Mill Pulverisette 7 with 800 rpm, even when adsorbed on a solid support (alumina, entries 29 and 30) or by using 10 eq of the substituted imidazole (entry 31). Neutral alumina was chosen based on a recent work of Salami, Krause, and co-workers [22] concerning the mechanochemical-assisted Passerini reactions where the alumina additive played a crucial role in increasing considerably the yield of the multicomponent reaction. Moreover, at 25 Hz, reactions of ethyl piperidine imidazole or pyridylmethyl imidazole with benzyl chloride provided the corresponding imidazolium salts **10** and **11** with conversions of 74% and 43% and yields of 67% and 81%, respectively (entries 32 and 33). More importantly, the reaction carried out in the Planetary Mill Pulverisette 7 (P7) at 800 rpm between 3,5-bis-trifluorophenyl imidazole and 2-bromomethyl pyridine afforded the corresponding imidazolium salt **12** with an estimated conversion of 70% and a yield of 72% (entry 34).

#### Aging

Considering the reactions performed for mechanochemical synthesis of substituted imidazoles and imidazolium salts, we observed in many cases, when the reaction was left for some days before purification, that conversions just after the grinding process were lower than the final yields. We attributed this to the so-called aging effect phenomenon. This effect and the advancement of the reaction analyzed by ^1^H NMR has been studied in three cases.

Table 4 summarizes the experiments where imidazolium salts were obtained after the grinding process developed previously, followed by storage of the reaction mixture in the jars for 10 days before purifying it. Conversions were determined by ^1^H-NMR after the 4 × 45 (5′) cycles and the yields are those obtained after 10 days of “aging”. The aging was performed by leaving the jars in a controlled room temperature box.

**Figure 1:**
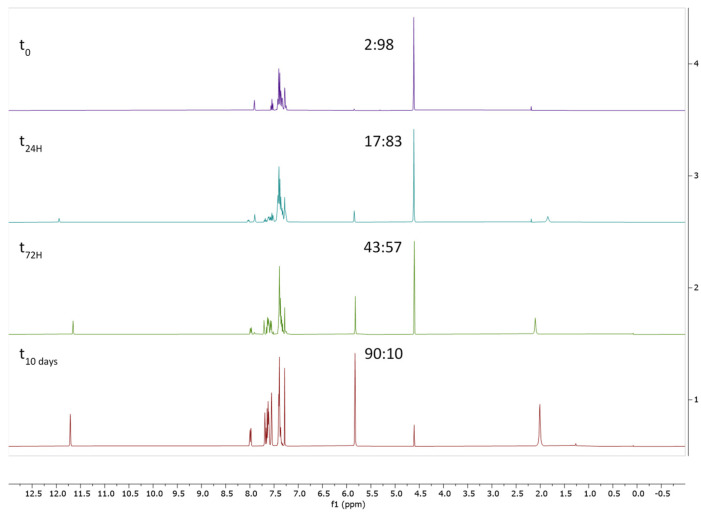
^1^H-NMR spectra showing the reaction during aging in the case of **8**.

Figure 1 illustrates the aging process in the case of imidazolium **8** (Table 4, entry 37) by ^1^H NMR follow-up. It is noteworthy that after grinding, the ratio between the final imidazolium salt and the starting substituted imidazole as determined by the integrations of CH_2_ protons of the benzyl groups is 2/98, while at the end of the aging process, the ratio is completely reversed (90/10). In comparison to the “non-aging” experiments, the isolated yields for the imidazolium salts under the same reaction conditions increased for **6** from 26% to 44%, and for **8** from 41% to 54% and from 13% to 48%.

Solvent-free reactions between solids or solids and liquids can take place spontaneously, but also be continued without (or with mild) energy impetus i.e., through vapor digestion, vapor-assisted aging, addition of catalysts, brief grinding, etc. [23].

The first example in human history is the description from Theophrastus of Ephesus (315 B.C.), as a student of Aristotle, in “De Lapidibus” or “On stones” [24]. Even if post-grinding aging processes (without any energy inputs) may proceed over hours or days, they usually lead to higher conversions and better yields of final compounds. This is what we observe in our case.

### 2.3. Synthesis of Neutral Complexes of Type NHCAuCl

**Scheme 3:**
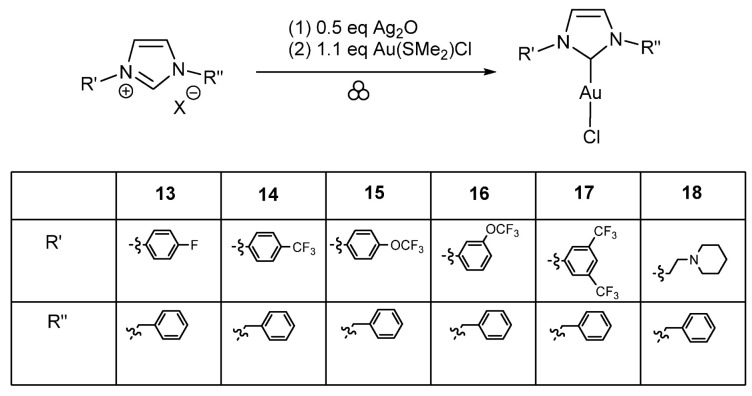
Synthesis of NHCAuCl complexes **13** to **18**. The grinding conditions are mentioned in Table 5.

The transmetalation reactions (Scheme 3) examined first (one-pot two-steps reactions) have been conducted in the Mixer Mill MM400 (Retsch) equipped with two 5 mL stainless steel, each jar with two balls of 5 mm Ø and at working frequency of 25 Hz (except entry 44, 20 Hz), starting with 0.5 eq of Ag_2_O for 1 eq of imidazolium salt (entries 38 to 44). After formation of the silver carbene complexes, 1.1 eq of Au(SMe_2_)Cl have been added to form the desired neutral NHCAuCl complexes. For these reactions, the corresponding conversions cannot be calculated and only the yields after purification can be given. For **13**, the mono NHC complex has been isolated with a yield of 63% and the raw data showed the formation of a small amount of cationic [AubisNHC]^+^ complex (entry 38). The neutral and cationic species could be distinguished due to the chemical shift of the carbene-carbon atom in the ^13^C NMR spectrum. In the case of neutral NHCAuCl complexes, the signal is at 170 ppm, while for the cationic [AubisNHC]^+^ complexes, it is about 180 ppm. The same has been observed in the case of **14**, giving a yield of 41% for the desired complex (entry 39). Complexes **15** with a p-OCF_3_ group on the phenyl system resulted in a yield of 6% (entry 40) while the isomer **16** bearing an m-OCF_3_ group resulted in a yield of 50% under these conditions (entry 41). The neutral NHCAuCl complex **17** has been obtained with a 54% yield (entry 42). Concerning the neutral complex **18**, it could be isolated with a yield of 83% and only a small amount of the cationic species has been formed (entry 43). Decreasing the frequency to 20 Hz resulted in lower yields (38%) in comparison to entry 38 for the formation of **13**, even by increasing the reaction time for the first step to 2 h (entry 44).

### 2.4. Synthesis of Cationic Complexes of Type AubisNHC:

**Scheme 4:**
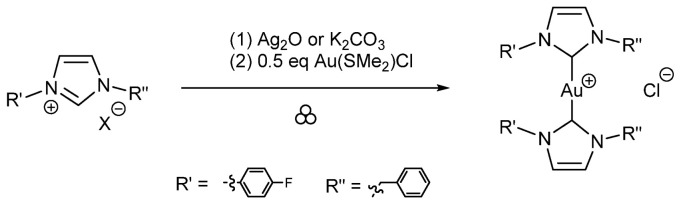
Synthesis of complex **19**.

These one-pot two-steps reactions have been conducted in the Mixer Mill MM400 (Retsch) equipped with two 5 mL stainless steel, each jar with two balls of 5 mm Ø and at working frequency of 20 Hz. For the synthesis of [AubisNHC]^+^ complex **19**, two strategies have been employed (Scheme 4, Table 6), the transmetalation route (entry 45) and the direct metalation (entries 46 to 48). Transmetalation resulted in a very good yield of 84%. For the direct metalation, the best result was obtained by reacting 1 equivalent of the base K_2_CO_3_ during 30 min before adding 0.5 eq of Au(SMe_2_)Cl, affording compound **19** in 73% yield (entry 48).

## 3. Materials and Methods

All reagents were used as received from commercial suppliers. The fluorinated substituted imidazoles used for the synthesis of imidazolium salts **5**–**9** and **12** and gold complexes **13**–**17** have been prepared following the protocols from [20]. The classical synthesis and characterization of the following compounds have been reported in the literature before: **1** [25], **2** [26], **3** [27], **4** [28], **10** [19], **11** [29]. Mechanochemical experiments have been performed using a planetary ball-mill Planetary Micro Mill PULVERISETTE 7 premium line (Fritsch, Fellbach, Germany) or a vibratory ball-mill Mixer Mill MM400 (Retsch, Haan, Germany) with stainless steel jars. NMR spectra were recorded on a Bruker AVANCE III 400 spectrometer (400 MHz for ^1^H, 101 MHz for ^13^C, 376 MHz for ^19^F), a Bruker AVANCE III 300 spectrometer (300 MHz for ^1^H, 75 MHz for ^13^C, 282 MHz for ^19^F) (Bruker, Ettlingen, Germany), in chloroform-*d* (CDCl_3_), methanol-*d*_4_ (CD_3_OD), dimethylsulfoxyde-*d*_6_ (DMSO-*d*_6_), or acetonitrile-*d_3_* (CD_3_CN). The residual proton signal of the deuterated solvent was used as an internal reference relative to TMS using ^1^H or ^13^C chemical shifts of the solvent as a secondary standard: CDCl_3_ *δ* = 7.26 ppm for ^1^H and 77.16 ppm for ^13^C, CD_3_OD *δ* = 3.31 ppm for ^1^H and 49.00 ppm for ^13^C, DMSO-*d_6_ δ* = 2.50 ppm for ^1^H and 39.52 ppm for ^13^C, CD_3_CN *δ* = 1.94 ppm for ^1^H and 1.32 ppm for ^13^C. Data are reported as follows: chemical shifts (*δ*) reported in parts per million (ppm), multiplicity (described as follows: s, singlet; d, doublet; t, triplet; q, quadruplet; dd, doublet of doublet; ddd, doublet of doublet of doublet; m, multiplet), coupling constants (J) reported in Hertz (Hz) and integrations. All the ^1^H and ^13^C signals were assigned based on chemical shifts, spin–spin coupling constants, splitting patterns and signal intensities, and by using ^1^H-^1^H COSY45, ^1^H-^13^C HMBC and ^1^H-^13^C HSQC/HMQC experiments. Gradient-enhanced ^1^H COSY45 was realized and included 2 scans for per increment. ^1^H-^13^C correlation spectra using a gradient-enhanced HSQC/HMQC sequence (delay was optimized for ^1^*J*_CH_ of 145 Hz) was obtained with 2 scans per increment. Gradient-enhanced HMBC experiment was performed allowing 62.5 ms for long-range coupling evolution (8 scans were accumulated). Typically, 1024 t2 data points were collected for 256 t1 increments. Thin-layer chromatography plates were performed on Silica Gel UV254—1000 μm from Analtech Uniplates™ (Sigma-Aldrich, St. Louis, MO, USA). Silica gel technical grade 0.063–0.200 mm−60 Å was used for column chromatography.

The description of synthesis is limited to reactions with good conversions and isolated products. Details concerning the different entries can be found in the Supplementary Materials.

Procedure A: General procedure for the synthesis of imidazoles in the Planetary Micro Mill PULVERISETTE 7 premium line (Fritsch): Imidazole and halogenated reagent were placed in a 20 mL stainless steel grinding jar with five stainless steel balls (10 mm diameter). Sometimes a base (NaOH, K_2_CO_3_, or KOH), a catalyst (CuSO_4_ or Cu(0)) or a liquid-assisted grinding (LAG) was added. The jar was closed and subjected to grinding for four cycles of 45 min with 5 min break between each cycle with a speed of 800, 500, or 300 rpm (rotations per minute). The crude product was recovered with methanol and the solvent was evaporated under reduced pressure.

Procedure B: General procedure for the synthesis of imidazoles in the Mixer Mill MM400 (Retsch): Imidazole and halogenated reagent were placed in a 10 mL stainless steel grinding jar with two stainless steel balls (10 mm diameter). Sometimes copper(0) as catalyst or a liquid-assisted grinding (LAG) was added. The jar was closed and subjected to grinding for four cycles of 45 min with 5 min break between each cycle with a speed of 10 or 25 Hz. The crude product was recovered with methanol and the solvent was evaporated under reduced pressure.

Synthesis of Imidazoles **1** to **4**

Synthesis of **1**: Procedure A. For each reaction, the crude product was extracted with a 1:1 mixture of DCM/CHCl_3_ (30 mL), filtered through a celite pad and washed with 3 × 20 mL of this solvent mixture. The desired product was obtained, after purification by column chromatography on silica gel with a gradient mixture of DCM and methanol from (100:0) to (98:2) as eluent, as a white solid.

^1^H NMR (300 MHz, CDCl_3_) *δ* 7.58 (s, 1H, H2), 7.43–7.30 (m, 3H, H9-H11), 7.21–7.15 (m, 2H, H8, H12), 7.11 (t, *J* = 1.1 Hz, 1H, H5), 6.92 (t, *J* = 1.3 Hz, 1H, H4), 5.14 (s, 2H, H6). ^13^C NMR (75 MHz, CDCl_3_) *δ* 137.5 (1C, C2), 136.2 (1C, C7), 129.8 (1C, C4), 129.0 (2C, C9, C11), 128.3 (1C, C10), 127.3 (2C, C8, C12), 119.3 (1C, C5), 50.8 (1C, C6).

Synthesis of **2**: Procedure A. For reactions with conversions higher than 50%, a liquid–liquid extraction was performed with DCM (15 mL) and water (20 mL). The organic phase was dried with MgSO_4_ and filtrated. The solvent was evaporated under vacuum, yielding a yellow oil.

^1^H NMR (400 MHz, CDCl_3_) δ 8.61 (ddd, J = 4.9, 1.8, 0.9 Hz, 1H, H11), 7.68 (td, J = 7.7, 1.8 Hz, 1H, H9), 7.63 (t, J = 1.1 Hz, 1H, H2), 7.28–7.23 (m, 1H, H10), 7.14 (t, J = 1.1 Hz, 1H, H5), 7.01 (t, J = 1.3 Hz, 1H, H4), 6.97 (dt, J = 7.9, 0.8 Hz, 1H, H8), 5.27 (s, 2H, H6). ^13^C NMR (75 MHz, CDCl_3_) δ 156.2 (1C, C7), 149.7 (1C, C11), 137.7 (1C, C2), 137.3 (1C, C9), 130.0 (1C, C8), 123.0 (1C, C10), 121.2 (1C, C4), 119.5 (1C, C5), 52.5 (1C, C6).

Synthesis of **3**: Procedures A or B.

^1^H NMR (300 MHz, CDCl_3_) δ 8.52 (ddd, *J* = 4.9, 1.9, 0.9 Hz, 1H, H10), 8.38 (s, 1H, H2), 7.85 (td, *J* = 8.2, 1.8 Hz, 1H, H8), 7.68 (s, 1H, H5), 7.39 (dt, *J* = 8.2, 1.0 Hz, 1H, H7), 7.28–7.24 (m, 1H, H9), 7.23 (s, 1H, H4).

Synthesis of **4**: Procedure A. For reactions with conversions higher than 50% a liquid–liquid extraction was performed with DCM (15 mL) and water (20 mL). The organic phase was dried with MgSO_4_ and filtrated. The solvent was evaporated under vacuum, yielding a yellow oil.

^1^H NMR (300 MHz, CDCl_3_) *δ* 7.54 (s, 1H, H5), 7.04 (s, 1H, H4), 6.98 (t, *J* = 1.2 Hz, 1H, H2), 4.03 (t, *J* = 6.2 Hz, 2H, H6), 2.65 (t, *J* = 6.7 Hz, 2H, H7), 2.42 (t, *J* = 5.3 Hz, 4H, H9,13), 1.59 (p, *J* = 5.7 Hz, 4H, H10,12), 1.50–1.39 (m, 2H, H11). ^13^C NMR (75 MHz, CDCl_3_) *δ* 137.4 (1C, C2), 129.2 (1C, C4), 119.2 (1C, C5), 59.5 (1C, C7), 54.7 (2C, C9,13), 44.9 (1C, C6), 26.0 (2C, C10,12), 24.2 (1C, C11).

Synthesis of Imidazolium Salts **5** to **12**

Purifications: Two different ways have been used to purify the product, either by precipitation or by column chromatography. Precipitation: the crude product was dissolved in 5 mL of DCM and precipitated with 15 mL of Et_2_O. The solid was filtered off and dried under vacuum to yield the desired product. Column chromatography: The crude product was purified by column chromatography on silica gel with a gradient mixture of DCM and methanol from (100:0) to (90:10) as eluent. The solvent was evaporated under reduced pressure to yield the desired imidazolium salt.

Synthesis of **5**: Procedure A—1-(4-(fluorophenyl)-1H-imidazole (1 eq) and benzyl chloride (1.1 eq), 800 rpm. Purification by precipitation, white solid.

^1^H NMR (400 MHz, CDCl_3_) *δ* 11.58 (t, *J* = 1.7 Hz, 1H, H2), 7.89–7.80 (m, 3H, H4,7,11), 7.67–7.57 (m, 3H, H5,8,10), 7.40–7.30 (m, 3H, H16,17,18), 7.24–7.14 (m, 2H, H15,19), 5.78 (s, 2H, H13). ^13^C NMR (75 MHz, CDCl_3_) *δ* 163.1 (d, *J* = 251.8 Hz, 1C, C9), 136.6 (1C, C2), 133.1 (1C, C14), 130.7 (d, *J* = 3.2 Hz, 1C, C6), 129.6 (1C, C17), 129.4 (2C, C16,18), 129.3 (2C, C15,19), 124.0 (d, *J* = 9.0 Hz, 2C, C7,11), 122.7 (1C, C5), 120.9 (1C, C4), 117.6 (d, *J* = 23.6 Hz, 2C, C8,10), 53.6 (1C, C13). ^19^F NMR (282 MHz, CDCl_3_) *δ* −109.36 (h, 1F). HMRS (ES^+^): Calc. for C_16_H_14_FN_2_ 253.1147, found 253.1141.

Synthesis of **6**: Procedure A—1-(4-(Trifluoromethyl)phenyl)-1H-imidazole (1 eq) and benzyl chloride (1.1 eq), 300 or 800 rpm. Purification by precipitation, white solid.

^1^H NMR (400 MHz, CDCl_3_): *δ* 11.53 (pseudot, *J* = 1.6 Hz, 1H, H2), 8.29 (t, *J* = 2.0 Hz, 1H, H4), 7.96 (d, *J* = 8.4 Hz, 2H, H7,11), 7.67 (t, *J* = 1.8 Hz, 1H, H5), 7.50–7.45 (d, *J* = 8.4 Hz, 2H, H8,10), 7.45–7.41 (m, 2H, H15,19), 7.11–7.04 (m, 3H, H16,17,18), 5.57 (s, 2H, H13). ^13^C NMR (101 MHz, CDCl_3_): *δ* 136.9 (1C, C6), 136.0 (1C, C2), 132.9 (1C, C14), 131.5 (q, *J* = 33.4 Hz, 1C, C9), 129.4 (1C, C17), 129.1 (2C, C16,18), 129.0 (2C, C15,19), 127.4 (q, *J* = 3.7 Hz, 2C, C8,10), 123.2 (1C, C5), 123.0 (q, *J* = 272.7 Hz, 1C, C12), 121.8 (2C, C7,11), 121.2 (1C, C4), 53.2 (1C, C13). ^19^F NMR (376 MHz, CDCl_3_): *δ* −63.04 (3F). HMRS (ES^+^): Calc. for C_17_H_14_F_3_N_2_ 303.1112, found 303.1109.

Synthesis of **7**: Procedure A—1-(4-(Trifluoromethoxy)phenyl)-1H-imidazole (1 eq) and benzyl chloride (1.1 eq), 800 rpm. Purification by precipitation, white solid.

^1^H NMR (400 MHz, CDCl_3_) *δ* 11.83 (s, 1H, H2), 7.94 (d, *J* = 8.5 Hz, 2H, H7,11), 7.70 (s, 1H, H4), 7.65–7.58 (m, 2H, H8,10), 7.47 (s, 1H, H5), 7.45–7.37 (m, 5H, H15,16,17,18,19), 5.80 (s, 2H, H13). ^13^C NMR (75 MHz, CDCl_3_) *δ* 150.0 (1C, C9), 137.1 (1C, C6), 132.8 (1C, C2), 132.7 (1C, C14), 129.7 (1C, C17), 129.5 (2C, C16,18), 129.4 (2C, C15,19), 123.6 (2C, C7,11), 122.8 (2C, C8,10), 122.7 (1C, C5), 120.6 (1C, C4), 120.2 (q, *J* = 259.3 Hz, 1C, C12), 53.9 (1C, C13). ^19^F NMR (282 MHz, CDCl_3_) *δ* −57.97 (3F). HMRS (ES^+^): Calc. For C_17_H_14_F_3_N_2_O, 319.1063, found 319.1058.

Synthesis of **8**: Procedure B—1-(3-(Trifluoromethoxy)phenyl)-1H-imidazole (1 eq) and benzyl chloride (1.1 eq), 10 or 25 Hz. Purification by precipitation or column chromatography, white solid.

^1^H NMR (400 MHz, CDCl_3_) δ 11.62 (t, *J* = 1.6 Hz, 1H, H2), 8.00 (t, *J* = 1.9 Hz, 1H, H4), 7.91 (ddd, *J* = 8.2, 2.3, 0.9 Hz, 1H, H9), 7.81 (t, *J* = 1.8 Hz, 1H, H5), 7.69–7.60 (m, 3H, H11,15,19), 7.53 (t, *J* = 8.3 Hz, 1H, H10), 7.31–7.25 (m, 4H, H7,16,17,18), 5.77 (s, 2H, H13). ^13^C NMR (101 MHz, CDCl_3_) δ 150.0 (d, *J* = 2.1 Hz), 136.5 (1C, C8), 135.6 (1C, C6), 133.1 (1C, C2), 132.1 (1C, C14), 129.5 (1C, C10), 129.3 (1C, C17), 129.3 (2C, C16,18), 123.4 (2C, C15,19), 122.0 (1C, C5), 120.9 (1C, C11), 120.4 (1C, C4), 120.2 (q, *J* = 259.4 Hz, 1C, C12), 114.6 (1C, C9), 53.5 (1C, C7). ^19^F NMR (282 MHz, CDCl_3_) δ −57.86 (3F). HMRS (ES^+^): Calc. For C_17_H_14_F_3_N_2_O 319.1063_,_ found 319.1058.

Synthesis of **10**: Procedure B—4-(2-(1H-imidazol-1-yl)ethyl)piperidine (1 eq) and benzyl chloride (1.1 eq), 25 Hz. Purification by column chromatography, white solid.

^1^H NMR (400 MHz, CDCl_3_) *δ* 10.78 (s, 1H), 7.58 (t, *J* = 1.6 Hz, 1H), 7.44–7.37 (m, 5H), 7.16 (t, *J* = 1.6 Hz, 1H), 5.57 (s, 2H), 4.55–4.39 (m, 2H), 2.89–2.72 (m, 2H), 2.46 (t, *J* = 5.3 Hz, 4H), 1.53 (m, 4H), 1.48–1.35 (m, 2H). ^13^C NMR (75 MHz, DMSO-*d*_6_): *δ* 137.7 (1C, C2), 135.0 (1C, benzyl), 129.4 (2C, benzyl), 129.2 (1C, benzyl), 128.9 (2C, benzyl), 123.3 (1C, C5), 123.2 (1C, C4), 54.6 (1C, C6), 52.8 (2C, C10), 52.5 (1C, C7), 43.5 (1C, C8), 22.4 (2C, C11), 21.7 (1C, C12).

Synthesis of **11**: Procedure B—2-(1H-imidazol-1-ylmethyl)pyridine (1 eq) and benzyl chloride (1.1 eq), 25 Hz. Purification by column chromatography, brown oil.

^1^H NMR (300 MHz, CDCl_3_) *δ* 11.13 (s, 1H, H2), 8.54 (dd, *J* = 4.8, 1.7 Hz, 1H, H11), 7.87–7.69 (m, 2H, H8,9), 7.59 (d, *J* = 1.8 Hz, 1H, H5), 7.48–7.36 (m, 5H, H15,16,17,18,19), 7.34–7.24 (m, 1H, H10), 7.15 (d, *J* = 1.8 Hz, 1H, H4), 5.77 (s, 2H, H6), 5.52 (s, 2H, H13).

Synthesis of **12**: Procedure A—1-(3,5-Bis(trifluoromethyl)phenyl)-1H-imidazole (1 eq) and 2-bromomethylpyridine hydrobromide (1 eq), 800 rpm. Purification by column chromatography, orange solid.

^1^H NMR (400 MHz, CD_3_OD) *δ* 10.02 (t, *J* = 1.7 Hz, 1H, H2), 8.74 (dq, *J* = 5.2, 0.9 Hz, 1H, H18), 8.55–8.52 (m, 2H, H7, H11), 8.37 (t, *J* = 1.9 Hz, 1H, H5), 8.33–8.30 (m, 1H, H9), 8.20 (td, *J* = 7.8, 1.8 Hz, 1H, H16), 8.02 (t, *J* = 1.7 Hz, 1H, H4), 7.84 (dt, *J* = 7.9, 1.0 Hz, 1H, H15), 7.69 (ddd, *J* = 7.8, 5.2, 1.1 Hz, 1H, H17), 5.87 (s, 2H, H13). ^13^C NMR (101 MHz, CD_3_OD) *δ* 150.4 (s, 2C, C7, C11), 146.9 (s, 1C, C18), 142.0 (s, 1C, C2), 137.7 (s, 1C, C16), 136.5 (s, 1C, C14), 133.3 (q, *J* = 34.5 Hz, 2C, C8, C10), 125.5 (s, 1C, C5), 125.0 (s, 1C, C4), 124.0 (d, *J* = 4.3 Hz, C6), 123.6 (hept, *J* = 3.9 Hz, C9), 122.6 (q, *J* = 272.6 Hz, 2C, C12, C12′), 122.4 (d, *J* = 5.5 Hz, C17), 52.3 (s, 1C, C13). ^19^F NMR (282 MHz, CD_3_OD) *δ* −64.23 (6F). HMRS (ES^+^): Calc. For C_17_H_12_F_6_N_3_ 372.0935, found 372.0942.

Synthesis of gold complexes **13** to **19**

General procedure for metalation reactions: Imidazolium chloride and Ag_2_O (entries 38 to 45) or K_2_CO_3_ (entries 46 to 48) were placed in a 5 mL stainless steel grinding jar with two stainless steel balls (5 mm diameter). The jar was closed and subjected to grinding in the Mixer Mill MM400 (Retsch) for 30 min, 1 h or 2 h, in the vibratory ball mill operated at 20 or 25 Hz. After that time, Au(SMe_2_)Cl was added to the reaction mixture and the jar was closed and subjected to grinding a new time for 1 h or 2 h. The crude product was recovered with methanol and the solvent was evaporated under reduced pressure. The crude product was dissolved in 50 mL of DCM or CH_3_CN and filtered through a pad of celite. The solvent was evaporated under reduced pressure.

Purifications: Depending on the product, two different ways have been used to purify the product, either by precipitation or by column chromatography. Precipitation: the crude product was dissolved in 5 mL of DCM and precipitated with 15 mL of Et_2_O. The solid was filtered off and dried under vacuum to yield the desired product. Column chromatography: The crude product was purified by column chromatography on silica gel with a gradient mixture of DCM and methanol from (100:0) to (90:10) as eluent. The solvent was evaporated under reduced pressure to yield the desired complex.

Synthesis of complex **13**: **5** (1 eq), Ag_2_O (0.5 eq) and Au(SMe_2_)Cl (1.1 eq), 20 or 25 Hz. Purification by column chromatography, white solid.

^1^H NMR (400 MHz, CDCl_3_) δ 7.65–7.59 (m, 2H, H7,11), 7.43–7.36 (m, 5H, H15,16,17,18,19), 7.22–7.17 (m, 2H, H8,10), 7.16 (d, *J* = 2.0 Hz, 1H, H4), 7.04 (d, *J* = 2.0 Hz, 1H, H5), 5.48 (s, 2H, H13). ^13^C NMR (101 MHz, CDCl_3_) δ 171.0 (1C, C2), 162.6 (d, *J* = 250.3 Hz, 1C, C9), 135.0 (d, *J* = 3.3 Hz, 1C, C6), 134.6 (1C, C14), 129.2 (2C, C16,18), 129.0 (1C, C17), 128.3 (2C, C15,19), 126.8 (d, *J* = 8.8 Hz, 2C, C7,11), 122.2 (1C, C5), 120.9 (1C, C4), 116.7 (d, *J* = 23.1 Hz, 2C, C8,10), 55.7 (1C, C13). ^19^F NMR (376 MHz, DMSO-d_6_) δ −112.59. HMRS (ES^+^): Calc. For C_18_H_16_AuFN_3_ (M^+^ + CH_3_CN) 490.1000, found 490.0994.

Synthesis of complex **14**: **6** (1 eq), Ag_2_O (0.5 eq) and Au(SMe_2_)Cl (1.1 eq), 25 Hz. Purification by column chromatography, white solid.

^1^H NMR (400 MHz, CDCl_3_) δ 7.90–7.77 (m, 4H, H7,8,10,11), 7.47–7.38 (m, 5H, H15,16,17,18,19), 7.25 (d, *J* = 2.0 Hz, 1H, H4), 7.11 (d, *J* = 2.0 Hz, 1H, H5), 5.52 (s, 2H, H13). ^13^C NMR (101 MHz, CDCl_3_) δ 171.2 (1C, C2), 141.6 (1C, C6), 134.3 (1C, C14), 131.5 (q, *J* = 33.2 Hz, 1C, C9), 129.3 (2C, C16,18), 129.1 (1C, C17), 128.4 (2C, C15,19), 127.1 (q, *J* = 3.8 Hz, 2C, C8,10), 125.2 (2C, C7,11), 123.4 (q, *J* = 272.7 Hz, 1C, C12), 121.8 (1C, C5), 121.4 (1C, C4), 55.9 (1C, C13). ^19^F NMR (282 MHz, DMSO-d_6_) δ −61.03. HMRS (ES^+^): Calc. For C_19_H_16_AuF_3_N_3_ (M^+^ + CH_3_CN) 540.0962, found 540.0963.

Synthesis of complex **15**: **7** (1 eq), Ag_2_O (0.5 eq) and Au(SMe_2_)Cl (1.1 eq), 25 Hz. Purification by column chromatography, white solid.

^1^H (400 MHz, CDCl_3_) δ 7.77–7.71 (m, 2H, H7,11), 7.46–7.40 (m, 5H, H15,16,17,18,19), 7.40–7.35 (m, 2H, H8,10), 7.21 (d, *J* = 2.0 Hz, 1H, H4), 7.08 (d, *J* = 2.0 Hz, 1H, H5), 5.52 (s, 2H, H13). ^13^C NMR (101 MHz, CDCl_3_) δ 171.0 (1c, C2), 149.4 (1C, C9), 137.2 (1C, C6), 134.4 (1C, C14), 129.3 (2C, C16,18), 129.1 (1C, C17), 128.3 (2C, C15,19), 126.4 (2C, C7,11), 122.1 (2C, C8,10), 122.1(1C, C5), 121.6 (1C, C12), 121.1 (1C, C4), 55.8(1C, C13). ^19^F NMR (376 MHz, DMSO-d_6_) δ −56.85 (3F). HMRS (DCI-CH_4_^+^): Calc. For C_17_H_13_AuF_3_N_2_O 515.0635, found 515.0646.

Synthesis of complex **16**: **8** (1 eq), Ag_2_O (0.5 eq) and Au(SMe_2_)Cl (1.1 eq), 25 Hz. Purification by column chromatography, white solid.

^1^H NMR (400 MHz, CDCl_3_) δ 7.75 (ddd, *J* = 8.1, 2.1, 0.9 Hz, 1H, H11), 7.58 (t, *J* = 8.2 Hz, 1H, H10), 7.51–7.46 (m, 1H, H7), 7.46–7.36 (m, 5H, H15,16,17,18,19), 7.37 (ddt, *J* = 7.3, 2.2, 1.1 Hz, 1H, H9), 7.23 (d, *J* = 2.0 Hz, 1H, H4), 7.09 (d, *J* = 2.1 Hz, 1H, H-5), 5.51 (s, 2H, H13). ^13^C NMR (101 MHz, CDCl_3_) δ 171.1 (1C, C2), 149.6 (d, *J* = 2.0 Hz, 1C, C8), 140.0 (1C, C6), 134.4 (1C, C14), 131.1 (1C, C10), 129.3 (2C, C16,18), 129.1 (1C, C17), 128.4 (2C, C15,19), 123.3 (1C, C9), 122.0 (1C, C5), 121.5 (1C, C4), 121.2 (1C, C11), 120.3 (q, *J* = 258.8 Hz, 1C, C12), 117.6 (1C, C7), 55.8 (1C, C13). ^19^F NMR (376 MHz, DMSO-d_6_) δ −56.77 (3F). HMRS (DCI-CH_4_^+^): Calc. For C_17_H_13_AuF_3_N_2_O 515.0657, found 515.0646.

Synthesis of complex **17**: **9** (1 eq), Ag_2_O (0.5 eq) and Au(SMe_2_)Cl (1.1 eq), 25 Hz. Purification by column chromatography, white solid.

^1^H NMR (400 MHz, CDCl_3_) *δ* 8.73–8.55 (m, 1H), 8.19 (s, 2H), 8.02 (s, 1H), 7.85–7.74 (m, 1H), 7.63 (dt, *J* = 7.8, 1.2 Hz, 1H), 7.55 (s, 1H), 7.37–7.32 (m, 1H), 5.61 (d, *J* = 1.7 Hz, 2H). ^13^C NMR (101 MHz, CDCl_3_) *δ* 175.5 (s, 1C), 153.8 (s, 1C), 150.0 (s, 1C), 140.2 (s, 1C), 137.7 (s, 1C), 133.4 (q, *J* = 34.5 Hz, 1C), 125.3 (s, 1C), 123.9 (s, 1C), 123.5 (s, 1C), 123.1 (d, *J* = 8.8 Hz, 1C), 122.4 (d, *J* =272.37 Hz, 1C), 121.1 (s, 1C), 57.0 (s, 1C). ^19^F NMR (376 MHz, CDCl_3_) *δ* −62.93 (6F). HMRS (DCI-CH_4_^+^): Calc. For C_18_H_12_AuF_6_N_2_ 567.0568, found 567.0570.

Synthesis of complex **18**: **10** (1 eq), Ag_2_O (0.5 eq) and Au(SMe_2_)Cl (1.1 eq), 25 Hz. Purification by column chromatography, yellow solid.

^1^H NMR (300 MHz, CDCl_3_) *δ* 7.42–7.29 (m, 6H, H4,16,17,18,19,20), 6.88 (d, *J* = 1.9 Hz, 1H, H5), 5.37 (s, 2H, H14), 4.55 (t, *J* = 6.7 Hz, 2H, H6), 3.09 (t, *J* = 6.7 Hz, 2H, H7), 2.77 (t, *J* = 5.6 Hz, 4H, H9,13), 1.85–1.72 (m, 4H, H10,12), 1.61–1.50 (m, 2H, H11). ^13^C NMR (101 MHz, CDCl_3_) *δ* 170.9 (1C, C2), 134.8 (1C, C15), 129.2 (2C, C17,19), 128.9 (1C, C18), 128.0 (2C, C16,20), 122.5 (1C, C5), 120.3 (1C, C4), 57.8 (1C, C6), 55.3 (1C, C7), 54.4 (1C, C14), 29.7 (2C, C9,13), 24.3 (2C, C10,12), 22.9 (1C, C11). HMRS (ES^+^): Calc. For C_17_H_23_AuN_3_^+^ 466.1556, found 466.1551.

Synthesis of complex **19**: Transmetalation reaction: **5** (1 eq), Ag_2_O (0.5 eq) and Au(SMe_2_)Cl (0.5 eq), 20 Hz. Purification by column chromatography, white solid. Direct metalation reaction: 5 (1 eq), K_2_CO_3_ (0.6 or 1 eq) for 30 min or 1h and Au(SMe_2_)Cl (0.5 eq) for 1 or 2 h, 20 Hz. Purification by precipitation, white solid.

^1^H NMR (400 MHz, CDCl_3_) *δ* 7.57–7.49 (m, 4H, H7,11), 7.36 (d, *J* = 2.0 Hz, 2H, H4), 7.35–7.32 (m, 6H, H16,17,18), 7.31 (d, *J* = 1.9 Hz, 2H, H5), 7.27–7.18 (m, 4H, H15,19), 7.06–6.98 (m, 4H, H8,10), 5.38 (s, 4H, H13).^13^C NMR (75 MHz, CDCl_3_) *δ* 182.2 (2C, C2), 162.5 (d, *J* = 250.4 Hz, 2C, C9), 135.2 (2C, C14), 135.0 (d, *J* = 3.0 Hz, 2C, C6), 129.1 (4C, C16,18), 128.7 (2C, C17), 127.6 (4C, C15,19), 126.8 (d, *J* = 8.9 Hz, 4C, C7,11), 123.2 (2C, C4), 123.0 (2C, C5), 116.5 (d, *J* = 23.2 Hz, 2C, C8,10), 54.9 (2C, C13). ^19^F NMR (282 MHz, CDCl_3_) *δ* −110.93 (hept, 2F). HMRS (ES^+^): Calc. For C_32_H_26_AuF_2_N_4_^+^ 701.1791, found 701.1777.

## 4. Conclusions

In conclusion, our work on the mechanochemical approach in metal carbene chemistry yielded very promising results. The mechanochemical approach has been studied for the different synthetic steps to obtain substituted imidazoles, imidazolium salts, and NHC-gold(I) complexes.

The first step concerning the formation of substituted imidazoles works well to bind an aliphatic carbon atom to the imidazole, while for aromatic substituents more drastic conditions are necessary and only for 2-bromo pyridine with imidazole, acceptable reaction conditions could be found. We are continuing this work in order to find the right conditions for coupling aromatic systems. We obtained four substituted imidazoles with good to very good yields (29–99%).

For the synthesis of imidazolium salts, the second substitution reaction has thus been limited to aliphatic carbon atoms. This yielded correct results in general and eight imidazolium salts could be obtained, five of them with good yields ranging from 46 to 81%. It is important to notice that the reaction can follow the aging process giving rise to imidazolium salts in very good yields even by using less energy.

For the formation of gold(I) complexes, the stoichiometry of the imidazolium salts and the gold(I) precursor is important to form either neutral NHCAuCl complexes or cationic [AubisNHC]^+^ systems. In this study, six mono NHC complexes of type NHCAuCl have been synthesized, five of them with good to very good yields (41–83%). Moreover, while in the literature the mechanochemical synthesis of the very few examples of [AubisNHC]^+^ complexes have been reported by transmetallation reactions using Ag_2_O as base, in this study, one [AubisNHC]^+^ has been obtained through direct metallation using K_2_CO_3_ as base and compared to the transmetallation route also applied.

In our ongoing work, we will focus also on the aging process to be employed for the first substitution reactions and the formation of NHC–gold complexes.

## Figures and Tables

**Table 1:** Reaction conditions for the formation of benzyl substituted imidazole **1**. Conversions are estimated by ^1^H NMR spectroscopy base on CH_2_ protons of the benzyl group. Yields are isolated yields.

Entry	Imidazole	Benzyl Chloride	Additional Base	Reaction Time and rpm	Conversion [%]	Yields [%]
1	1 eq.	1 eq.	-	4 × 45 (5′) 800	46	18
2	2 eq.	1 eq.	-	4 × 45 (5′) 800	74	39
3	2.5 eq.	1 eq.	-	4 × 45 (5′) 800	76	73
4	5 eq.	1 eq.	-	4 × 45 (5′) 800	99	99
5	5 eq.	1 eq.	-	4 × 45 (5′) 500	95	92
6	1 eq.	1 eq.	1 eq. K_2_CO_3_	4 × 45 (5′) 800	83	43
7	1 eq.	1 eq.	1.6 eq. NaOH	4 × 45 (5′) 800	74	62
8	1 eq.	1 eq.	2 eq. KOH	4 × 45 (5′) 800	93	76

**Table 2:** Reaction conditions for the formation of substituted imidazoles **2** to **4**. Yields are isolated yields.

Entry	Compound	Imidazole	Halogenated Reagent	Additional Base	Reaction Time and rpm	Yields [%]
9	**2**	5 eq.	1 eq. PyCH_2_Br·HBr		4 × 45 (5′) 800	-
10	**2**	1 eq.	1 eq. PyCH_2_Br·HBr	2 eq. KOH	4 × 45 (5′) 800	55
11	**2**	5 eq.	1 eq. PyCH_2_Br·HBr	LAG THF	4 × 45 (5′) 800	68
12	**2**	5 eq.	1 eq. PyCH_2_Br·HBr	1 eq. KOH, LAG THF	4 × 45 (5′) 800	72
13	**2**	1 eq.	1 eq. PyCH_2_Br·HBr	4 eq. KOH, LAG THF	4 × 45 (5′) 800	68
14	**3**	1 eq.	1 eq. PyBr	1 eq. KOH	4 × 45 (5′) 800	-
15	**3**	1 eq.	2 eq. PyBr	2 eq. KOH	4 × 45 (5′) 800	-
16	**3**	1 eq.	1 eq. PyBr	2 eq. K_2_CO_3_, 0.1 eq. CuSO_4_	4 × 45 (5′) 800	-
17	**3**	5 eq.	1 eq. PyBr	0.2 eq. Cu	4 × 45 (5′) 800	50
18	**3**	5 eq.	1 eq. PyBr	1 eq. KOH + 0.2 eq. Cu	4 × 45 (5′) 800	37
19	**3**	5 eq.	1 eq. PyBr	0.2 eq. Cu, LAG THF	4 × 45 (5′) 25 (800)	29 (27)
20	**4**	5 eq.	1 eq. PipEtCl·HCl		4 × 45 (5′) 800	64
21	**4**	5 eq.	1 eq. PipEtCl·HCl	1 eq. KOH	4 × 45 (5′) 800	64

**Table 3:** Reaction conditions for the formation of imidazolium salts **5** to **12**. Conversions are estimated by ^1^H NMR spectroscopy based on CH_2_ protons of the benzyl or PyCH_2_ groups. Yields are isolated yields.

Entry	Imidazolium Salt	R′	R″	Equivalence Imidazole-R′:R″-X	rpm or Hz	Conversion [%]	Yield [%]
22	**5**	*p*-F	Bn	1:1.1	800	42	18
23	**6**	*p*-CF_3_	Bn	1:1.1	800	69	60
24	**6**	*p*-CF_3_	Bn	1:1.1	300	35	26
25	**7**	*p*-OCF_3_	Bn	1:1.1	800	46	46
26	**8**	*m*-OCF_3_	Bn	1:1.1	25	42	41
27	**8**	*m*-OCF_3_	Bn	1:1.1	10	7	13
28	**9**	Bis-CF_3_	Bn	1:1.1	25	0	-
29	**9**	Bis-CF_3_	Bn	1:1.1	800	0	-
30	**9**	Bis-CF_3_	Bn	1:1.1	800 with alumina	0	-
31	**9**	Bis-CF_3_	Bn	1:10	800	5	-
32	**10**	PipEt	Bn	1:1.1	25	74	67
33	**11**	PyCH_2_	Bn	1:1.1	25	43	81
34	**12**	Bis-CF_3_	PyCH_2_	1:1	800	70	72

**Table 4:** Reaction conditions for the aging experiments to form imidazolium salts **6** and **8**. Conversions are estimated by ^1^H NMR spectroscopy based on CH_2_ protons of the benzyl or PyCH_2_ groups. Yields are isolated yields.

Entry	Imidazolium Salt	R′	R″	Equivalence Imidazole-R′:R″-X	rpm or Hz	Conversion * [%]	Yields [%]
35	**6**	*p*-CF_3_	Bn	1:1.1	300 with aging	11 (68)	44
36	**8**	*m*-OCF_3_	Bn	1:1.1	25 with aging	20 (90)	54
37	**8**	*m*-OCF_3_	Bn	1:1.1	10 with aging	2 (90)	48

* Conversions in brackets are obtained after the aging process. The yields indicated are after aging and purification.

**Table 5:** Reaction conditions for the formation of NHC–gold(I) complexes **13** to **19**. All reactions have been performed with 25 Hz except entry 44.

Entry	Complex	R′	R″	Base Reaction Time	Au(SMe_2_)Cl Reaction Time	NHCAuCl Yield [%]
38	**13**	*p*-F	Bn	0.5 eq. Ag_2_O 30′	1.1 eq. 1 h	63
39	**14**	*p*-CF_3_	Bn	0.5 eq. Ag_2_O 30′	1.1 eq. 1 h	41
40	**15**	*p*-OCF_3_	Bn	0.5 eq. Ag_2_O 30′	1.1 eq. 1 h	6
41	**16**	*m*-OCF_3_	Bn	0.5 eq. Ag_2_O 30′	1.1 eq. 1 h	50
42	**17**	Bis-CF_3_	Bn	0.5 eq. Ag_2_O 30′	1.1 eq. 1 h	54
43	**18**	PipEt	Bn	0.5 eq. Ag_2_O 2 h	1.1 eq. 1 h	83
44	**13**	*p*-F	Bn	0.5 eq. Ag_2_O 2 h	1.2 eq. 1 h with 20 Hz	38

**Table 6:** Reaction conditions for the formation of gold(I) complex **19**. All reactions have been performed with 20 Hz.

Entry	Complex	R′	R″	Base, Reaction Time	Au(SMe_2_)Cl, Reaction Time	[AubisNHC]X Yields [%]
45	**19**	*p*-F	Bn	0.5 eq Ag_2_O, 30′	0.5 eq., 1 h	84
46	**19**	*p*-F	Bn	0.6 eq. K_2_CO_3_, 30′	0.5 eq., 1 h	36
47	**19**	*p*-F	Bn	1.2 eq. K_2_CO_3_, 1 h	0.5 eq., 2 h	43
48	**19**	*p*-F	Bn	1 eq. K_2_CO_3_, 30′	0.5 eq. 1 h	73

## Data Availability

All data created during this study are presented in the manuscript or in the Supplementary Materials.

## Supplementary Materials

The following supporting information can be downloaded at: https://www.mdpi.com/article/10.3390/molecules30030522/s1, experimental details and NMR spectra, Figures S1 to S41 with Figure S1: ^1^H NMR spectrum of **1**, Figure S2: ^13^C NMR spectrum of **1**, Figure S3: ^1^H NMR spectrum of **2**, Figure S4: ^13^C NMR spectrum of **2**, Figure S5: ^1^H NMR spectrum of **3**, Figure S6: ^1^H NMR spectrum of **4**, Figure S7: ^13^C NMR spectrum of **4**, Figure S8: ^1^H NMR spectrum of **5**, Figure S9: ^13^C NMR spectrum of **5**, Figure S10: ^19^F NMR spectrum of **5**, Figure S11: ^1^H NMR spectrum of **6**, Figure S12: ^13^C NMR spectrum of **6**, Figure S13: ^19^F NMR spectrum of **6**, Figure S14: ^1^H NMR spectrum of **7**, Figure S15: ^13^C NMR spectrum of **7**, Figure S16: ^19^F NMR spectrum of **7**, Figure S17: ^1^H NMR spectrum of **8**, Figure S18: ^13^C NMR spectrum of **8**, Figure S19: ^19^F NMR spectrum of **8**, Figure S20: ^1^H NMR spectrum of **10**, Figure S21: ^1^H NMR spectrum of **11**, Figure S22: ^1^H NMR spectrum of **12**, Figure S23: ^13^C NMR spectrum of **12**, Figure S24: ^19^F NMR spectrum of **12**, Figure S25: ^1^H NMR spectrum of **13**, Figure S26: ^13^C NMR spectrum of **13**, Figure S27: ^19^F NMR spectrum of **13**, Figure S28: ^1^H NMR spectrum of **14**, Figure S29: ^13^C NMR spectrum of **14**, Figure S30: ^19^F NMR spectrum of **14**, Figure S31: ^1^H NMR spectrum of **15**, Figure S32: ^13^C NMR spectrum of **15**, Figure S33: ^19^F NMR spectrum of **15**, Figure S34: ^1^H NMR spectrum of **16**, Figure S35: ^13^C NMR spectrum of **16**, Figure S36: ^19^F NMR spectrum of **16**, Figure S37: ^1^H NMR spectrum of **17**, Figure S38: ^1^H NMR spectrum of **18**, Figure S39: ^1^H NMR spectrum of **19**, Figure S40: ^13^C NMR spectrum of **19**, Figure S41: ^19^F NMR spectrum of **19**.
