# Supplementary material for: Mechanochemistry to Form Substituted Imidazoles, Imidazolium Salts and NHC–Gold Complexes with Fluorine-Containing Groups
Source: Molecules. 2025 Jan 24;30(3):522. doi: 10.3390/molecules30030522 (PMC11820496; doi:10.3390/molecules30030522)
Supplement: Supplementary file 1 [file molecules-30-00522-s001.zip › molecules-3409373-supplementary.pdf]

Supporting Information

Mechanochemistry to Form Substituted Imidazoles, Imidazolium Salts and NHC-Gold-Complexes with Fluorine-Containing Groups

Chloé Salis, Sabrina Mohammedi, Lucia Turazza, Yuna Blandin, Maritie Garnier, Catherine Hemmert, Michel Baltas,* Heinz Gornitzka*

Synthetic detail for **1**-**19**	Pages 2-5
NMR spectra of **1**-**19**	Pages 6-26

Synthesis of **1:**

Entry 1: Imidazole (339 mg, 5.0 mmol, 1 eq) and benzyl chloride (693 mg, 5.5 mmol, 1 eq), yield 174 mg (18%).

Entry 2: Imidazole (170 mg, 2.50 mmol, 2 eq) and benzyl chloride (158 mg, 1.25 mmol, 1 eq), yield 78 mg (39%).

Entry 3: Imidazole (585 mg, 8.60 mmol, 2.5 eq) and benzyl chloride (435 mg, 3.44 mmol, 1 eq), yield 399 mg (73%).

Entry 4: Imidazole (627 mg, 9.20 mmol, 5 eq) and benzyl chloride (233 mg, 1.84 mmol, 1 eq), yield 287 mg (99%).

Entry 5: Imidazole (627 mg, 9.20 mmol, 5 eq) and benzyl chloride (233 mg, 1.84 mmol, 1 eq), yield 268 mg (92%).

Entry 6: Imidazole (68 mg, 1 mmol, 1 eq), benzyl chloride (127 mg, 1 mmol, 1 eq) and K_2CO_3 (138 mg, 1 mmol, 1 eq), yield 68 mg (43%).

Entry 7: Imidazole (204 mg, 3.00 mmol, 1 eq), benzyl chloride (456 mg, 3.60 mmol, 1.2 eq) and NaOH (198 mg, 4.95 mmol, 1.65 eq), yield 292 mg (62%).

Entry 8: Imidazole (225 mg, 3.30 mmol, 1 eq), benzyl chloride (459 mg, 3.63 mmol, 1.1 eq) and KOH (370 mg, 6.6 mmol, 2 eq), yield 397 mg (76%).

Synthesis of **2:**

Entry 10: Imidazole (69 mg, 0.41 mmol, 1 eq), 2-(bromomethyl)pyridine hydrobromide (103 mg, 0.41 mmol, 1 eq) and KOH (46 mg, 0.82 mmol, 2 eq), yield 26 mg (55%).

Entry 11: Imidazole (340 mg, 5 mmol, 5 eq), 2-(bromomethyl)pyridine hydrobromide (253 mg, 1 mmol, 1 eq) and 297 µL of THF as LAG, yield 109 mg (68%).

Entry 12: Imidazole (335 mg, 4.90 mmol, 5 eq), 2-(bromomethyl)pyridine hydrobromide (249 mg, 0.98 mmol, 1 eq), KOH (55 mg, 0.98 mmol, 1 eq) and 319 µL of THF as LAG, yield 113 mg (72%).

Entry 13: Imidazole (82 mg, 1.20 mmol, 1 eq), 2-(bromomethyl)pyridine hydrobromide (304 mg, 1.20 mmol, 1 eq), KOH (270 mg, 4.80 mmol, 4 eq) and 328 µL of THF as LAG, yield 130 mg (68%).

Synthesis of **3:**

Entry 17: Procedure A - Imidazole (681 mg, 10 mmol, 5 eq), 2-bromopyridine (316 mg, 2 mmol, 1 eq) and copper(0) (25.4 mg, 0.40 mmol, 0.2 eq). The crude product was extracted with a 1:1 mixture of DCM/$CHCl_3$ (30 mL), filtered through a celite pad and washed with 3 x 20 mL of this solvent mixture. A liquid-liquid extraction was done with DCM (20 mL) and water (30 mL). The desired product was obtained after drying the organic phase with $MgSO_4$, filtration and drying under vacuum as a yellow oil (146 mg, 50% yield).

Entry 18: Procedure A - Imidazole (613 mg, 9 mmol, 5 eq), 2-bromopyridine (284 mg, 1.80 mmol, 1 eq), KOH (101 mg, 1.8 mmol, 1 eq) and copper(0) (23 mg, 0.36 mmol, 0.2 eq). The desired product was obtained after purification by column chromatography on silica gel with a gradient mixture of DCM and methanol from (100:0) to (98:2) as eluent. Yellow oil (96 mg, 37% yield).

Entry 19: Procedure A - Imidazole (453 mg, 6.65 mmol, 5 eq), 2-bromopyridine (210 mg, 1.33 mmol, 1 eq), copper(0) (17 mg, 0.27 mmol, 0.2 eq) and 340 µL of THF as LAG. The crude product was extracted with a 1:1 mixture of DCM/$CHCl_3$ (30 mL), filtered through a celite pad and washed with 3 x 20 mL of this solvent mixture. A liquid-liquid extraction was done with DCM (20 mL) and water (30 mL). The desired product was obtained, after drying the organic phase with $MgSO_4$, filtration and drying under vacuum, as a yellow oil (56 mg, 27% yield).

Entry 19: Procedure B - Imidazole (228 mg, 3.35 mmol, 5 eq), 2-bromopyridine (106 mg, 0.67 mmol, 1 eq), copper(0) (8 mg, 0.13 mmol, 0.2 eq) and 171 µL of THF as LAG. The crude product was extracted with a 1:1 mixture of DCM/$CHCl_3$ (30 mL), filtered through a celite pad and washed with 3 x 20 mL of this solvent mixture. A liquid-liquid extraction was done with DCM (20 mL) and water (30 mL). The desired product was obtained, after drying the organic phase with $MgSO_4$, filtration and drying under vacuum, as a yellow oil (25 mg, 29% yield).

Synthesis of **4:**

Entry 20: Imidazole (582 mg, 8.55 mmol, 5 eq) and 1-(2-chloroethyl)piperidine hydrochloride (315 mg, 1.71 mmol, 1 eq), four cycles of 45 min with 800 rpm, yield 195 mg (64%).

Entry 21: Imidazole (623 mg, 9.15 mmol, 5 eq), 1-(2-chloroethyl)piperidine hydrochloride (337 mg, 1.83 mmol, 1 eq) and KOH (103 mg, 1.83 mmol, 1 eq), four cycles of 45 min with 800 rpm, yield 210 mg (64%).

Synthesis of Imidazolium Salts 5 to 12

Purifications: Depending on the product, two different ways have been used to purify the product, either by *precipitation* or by *column chromatography*. *Precipitation*: the crude product was dissolved in 5 mL of DCM and precipitated with 15 mL of Et_2O. The solid was filtered off and dried under vacuum to give the desired product. *Column chromatography*: The crude product was purified by column chromatography on silica gel with a gradient mixture of DCM and methanol from (100 : 0) to (90 : 10) as eluent. The solvent was evaporated under reduced pressure to give the desired imidazolium salt.

Synthesis of **5:**

Entry 22. Procedure A - 1-(4-(fluorophenyl)-1H-imidazole (511 mg, 3.15 mmol, 1 eq) and benzyl chloride (439 mg, 3.47 mmol, 1.1 eq), 800 rpm. Purification by precipitation, white solid (165 mg, 18% yield).

Synthesis of **6:**

Entry 23. Procedure A - 1-(4-(Trifluoromethyl)phenyl)-1H-imidazole (573 mg, 2.70 mmol, 1 eq) and benzyl chloride (376 mg, 2.97 mmol, 1.1 eq), 800 rpm. Purification by precipitation, white solid (547 mg, 60% yield).

Entry 24: Procedure A - 1-(4-(Trifluoromethyl)phenyl)-1H-imidazole (573 mg, 2.70 mmol, 1 eq) and benzyl chloride (376 mg, 2.97 mmol, 1.1 eq), 300 rpm. Purification by precipitation, white solid (238 mg, 26% yield).

Entry 35: Procedure A - 1-(4-(Trifluoromethyl)phenyl)-1H-imidazole (573 mg, 2.70 mmol, 1 eq) and benzyl chloride (376 mg, 2.97 mmol, 1.1 eq), 300 rpm with aging. Purification by precipitation, white solid (406 mg, 44% yield).

Synthesis of **7:**

Entry 25: Procedure A - 1-(4-(Trifluoromethoxy)phenyl)-1H-imidazole (651 mg, 2.85 mmol, 1 eq) and benzyl chloride (397 mg, 3.14 mmol, 1.1 eq), 800 rpm. Purification by precipitation, white solid (470 mg, 46% yield).

Synthesis of **8:**

Entry 26: Procedure B - 1-(3-(Trifluoromethoxy)phenyl)-1H-imidazole (180 mg, 0.79 mmol, 1 eq) and benzyl chloride (110 mg, 0.87 mmol, 1.1 eq), 25 Hz. Purification by column chromatography, white solid (182 mg, 41% yield).

Entry 27: Procedure B - 1-(3-(Trifluoromethoxy)phenyl)-1H-imidazole (180 mg, 0.79 mmol, 1 eq) and benzyl chloride (110 mg, 0.87 mmol, 1.1 eq), 10 Hz. Purification by precipitation, white solid (44 mg, 13% yield).

Entry 36: Procedure B - 1-(3-(Trifluoromethoxy)phenyl)-1H-imidazole (217 mg, 0.95 mmol, 1 eq) and benzyl chloride (132 mg, 1.04 mmol, 1.1 eq), 25 Hz with aging. Purification by column chromatography, white solid (182 mg, 54% yield).

Entry 37: Procedure B - 1-(3-(Trifluoromethoxy)phenyl)-1H-imidazole (217 mg, 0.95 mmol, 1 eq) and benzyl chloride (132 mg, 1.04 mmol, 1.1 eq), 10 Hz with aging. Purification by precipitation, white solid (135 mg, 48% yield).

Synthesis of **10:**

Entry 32. Procedure B - 4-(2-(1H-imidazol-1-yl)ethyl)piperidine (90 mg, 0.50 mmol, 1 eq) and benzyl chloride (70 mg, 0.55 mmol, 1.1 eq), 25 Hz. Purification by column chromatography, white solid (102 mg, 67% yield).

Synthesis of **11:**

Entry 33. Procedure B - 2-(1H-imidazol-1-ylmethyl)pyridine (76 mg, 0.48 mmol, 1 eq) and benzyl chloride (67 mg, 0.53 mmol, 1.1 eq), 25 Hz. Purification by column chromatography, brown oil (111 mg, 81% yield).

Synthesis of **12:**

Entry 34. Procedure A - 1-(3,5-Bis(trifluoromethyl)phenyl)-1H-imidazole (743 mg, 2.65 mmol, 1 eq) and 2-bromomethylpyridine hydrobromide (670 mg, 2.65 mmol, 1 eq), 800 rpm. Purification by column chromatography, orange solid (863 mg, 72% yield).

Synthesis of gold complexes 13 to 19

Synthesis of complex **13:**

Entry 38: Transmetalation reaction, **5** (69 mg, 0.24 mmol, 1 eq), Ag_2O (30 mg, 0.13 mmol, 0.5 eq) and $Au(SMe_2)Cl$ (83 mg, 0.28 mmol, 1.1 eq), 25 Hz. Purification by column chromatography, white solid (73 mg, 63% yield).

Entry 44: Transmetalation reaction, **5** (29 mg, 0.10 mmol, 1 eq), Ag_2O (12 mg, 0,05 mmol, 0.5 eq) and $Au(SMe_2)Cl$ (34 mg, 0.12 mmol, 1.1 eq), 20 Hz. Purification by column chromatography, white solid (19 mg, 38% yield).

Synthesis of complex **14:**

Entry 39. Transmetalation reaction, **6** (81 mg, 0.22 mmol, 1 eq), Ag_2O (29 mg, 0.12 mmol, 0.5 eq) and Au(SMe_2)Cl (82 mg, 0.28 mmol, 1.1 eq), 25 Hz. Purification by column chromatography, white solid (52 mg, 41% yield).

Synthesis of complex **15:**

Entry 40. Transmetalation reaction, **7** (73 mg, 0.20 mmol, 1 eq), Ag_2O (26 mg, 0.11 mmol, 0.5 eq) and Au(SMe_2)Cl (76 mg, 0.26 mmol, 1.1 eq), 25 Hz. Purification by column chromatography, white solid (7 mg, 6% yield).

Synthesis of complex **16:**

Entry 41. Transmetalation reaction, **8** (78 mg, 0.22 mmol, 1 eq) Ag_2O (26 mg, 0.11 mmol, 0.5 eq) and Au(SMe_2)Cl (76 mg, 0.26 mmol, 1.1 eq), 25 Hz. Purification by column chromatography, white solid (61 mg, 50% yield).

Synthesis of complex **17:**

Entry 42. Transmetalation reaction, **9** (91 mg, 0.20 mmol, 1 eq), Ag_2O (24 mg, 0.10 mmol, 0.5 eq) and Au(SMe_2)Cl (68 mg, 0.23 mmol, 1.1 eq), 25 Hz. Purification by column chromatography, white solid (35 mg, 54% yield).

Synthesis of complex **18:**

Entry 43. Transmetalation reaction, **10** (22 mg, 0.07 mmol, 1 eq), Ag_2O (16 mg, 0.07 mmol, 0.5 eq) and Au(SMe_2)Cl (21 mg, 0,07 mmol, 1.1 eq), 25 Hz. Purification by column chromatography, yellow solid (29 mg, 83% yield).

Synthesis of complex **19:**

Entry 45: Transmetalation reaction, **5** (47 mg, 0.16 mmol, 1 eq), Ag_2O (19 mg, 0,08 mmol, 0.5 eq) and Au(SMe_2)Cl (24 mg, 0.08 mmol, 0.5 eq), 20 Hz. Purification by column chromatography, white solid (51 mg, 84% yield).

Entry 46: Direct metalation reaction, **5** (43 mg, 0.15 mmol, 1 eq), K_2CO_3 (13 mg, 0.09 mmol, 0.6 eq) for 30 min and Au(SMe_2)Cl (22 mg, 0.08 mmol, 0.5 eq) for one hour, 20 Hz. Purification by precipitation, white solid (20 mg, 36% yield).

Entry 47: Direct metalation reaction, **5** (43 mg, 0.15 mmol, 1 eq), K_2CO_3 (21 mg, 0.15 mmol, 1 eq) for one hour and Au(SMe_2)Cl (22 mg, 0.08 mmol, 0.5 eq) for two hours, 20 Hz. Purification by precipitation, white solid (24 mg, 43% yield).

Entry 48: Direct metalation reaction, **5** (43 mg, 0.15 mmol, 1 eq), K_2CO_3 (21 mg, 0.15 mmol, 1 eq) for 30 min and Au(SMe_2)Cl (22 mg, 0.08 mmol, 0.5 eq) for one hour, 20 Hz. Purification by precipitation, white solid (41 mg, 73% yield).

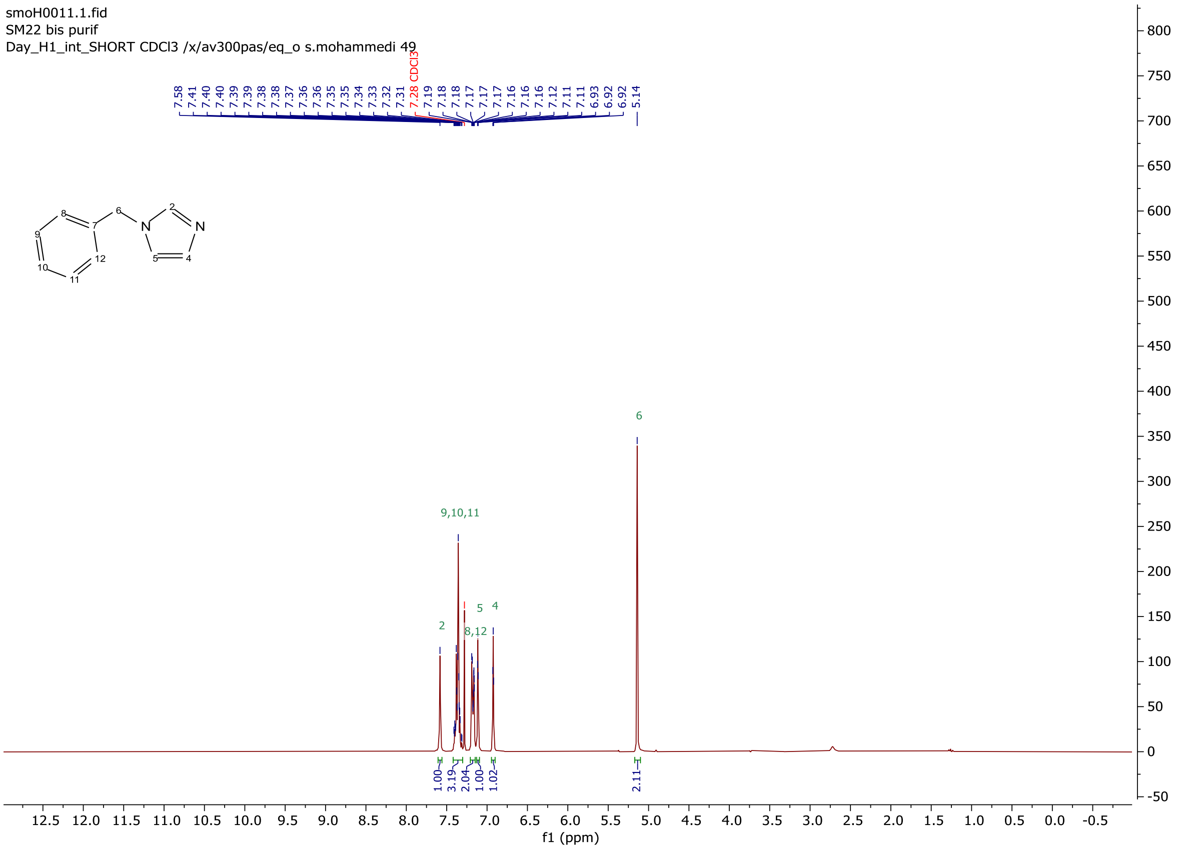

Figure S1: ^{1}H NMR spectrum of **1**.

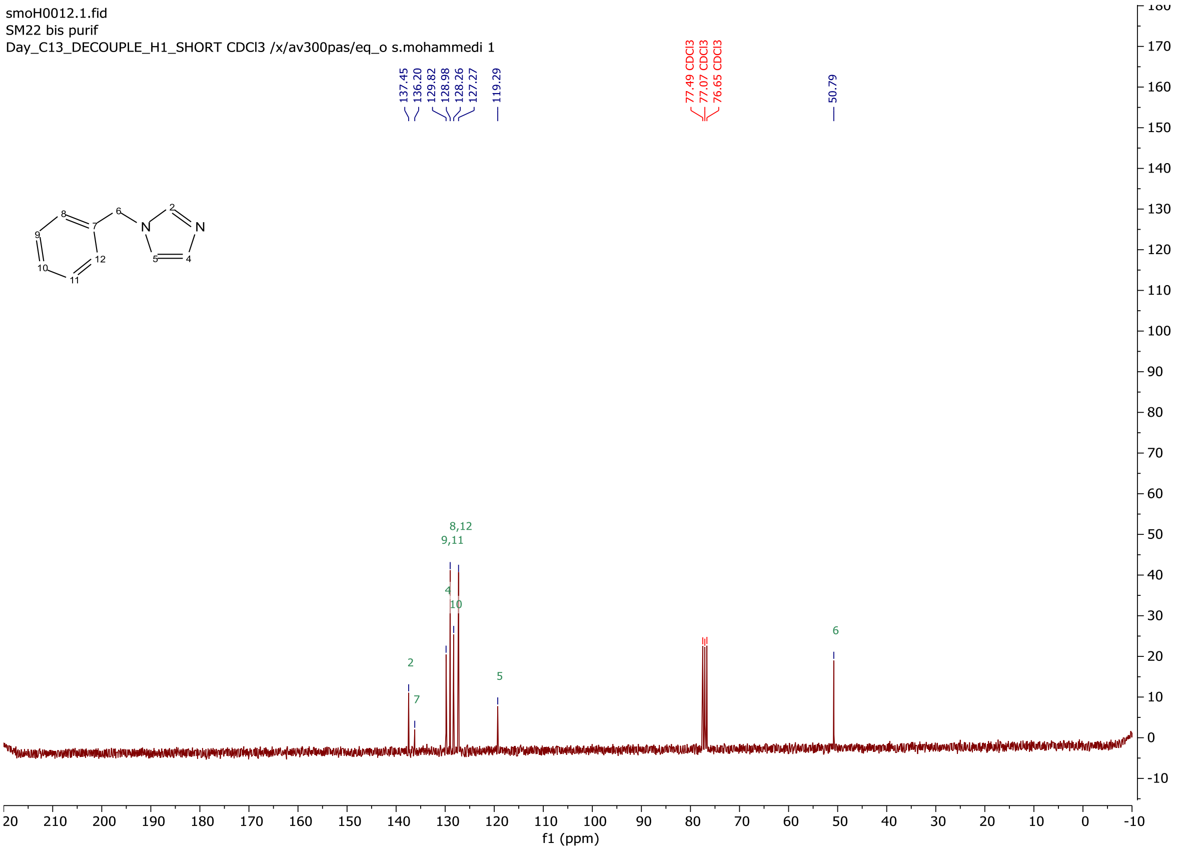

Figure S2: ^{13}C NMR spectrum of **1**.

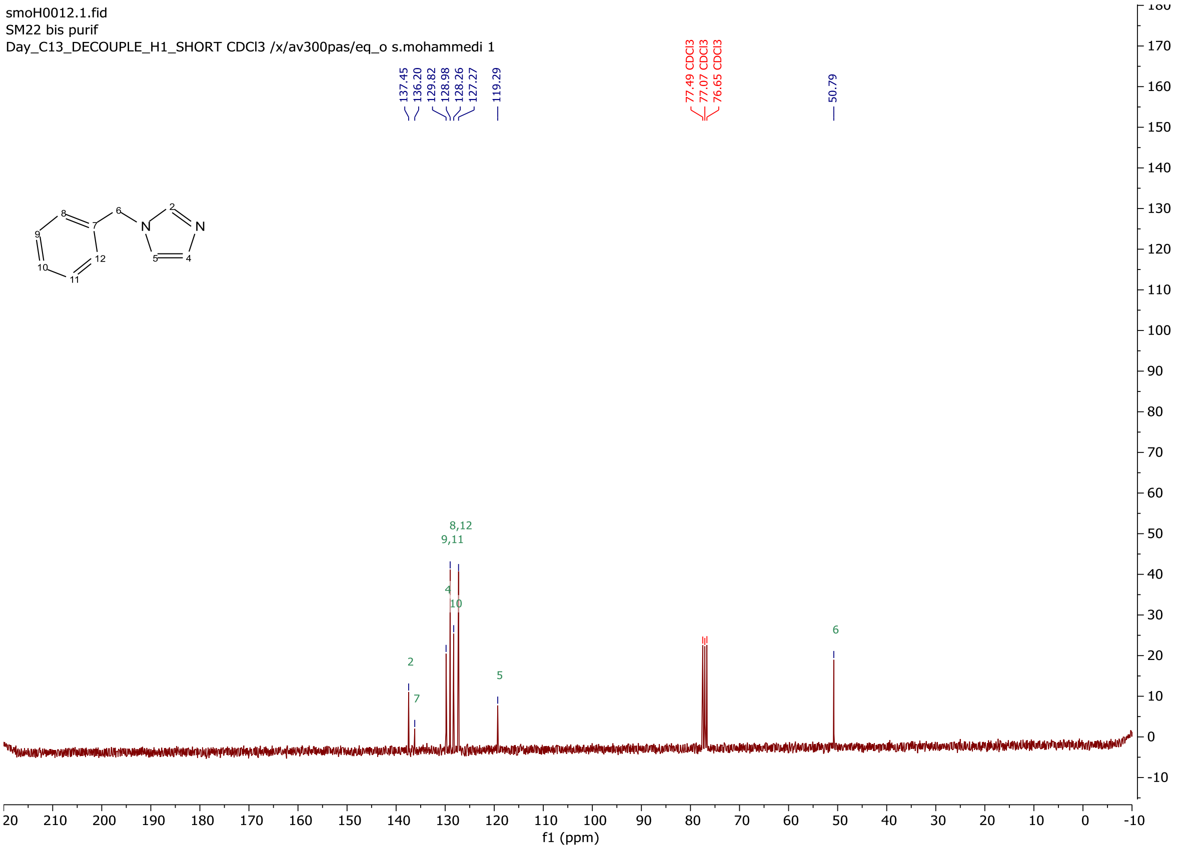

Figure S3: ^{1}H NMR spectrum of **2**.

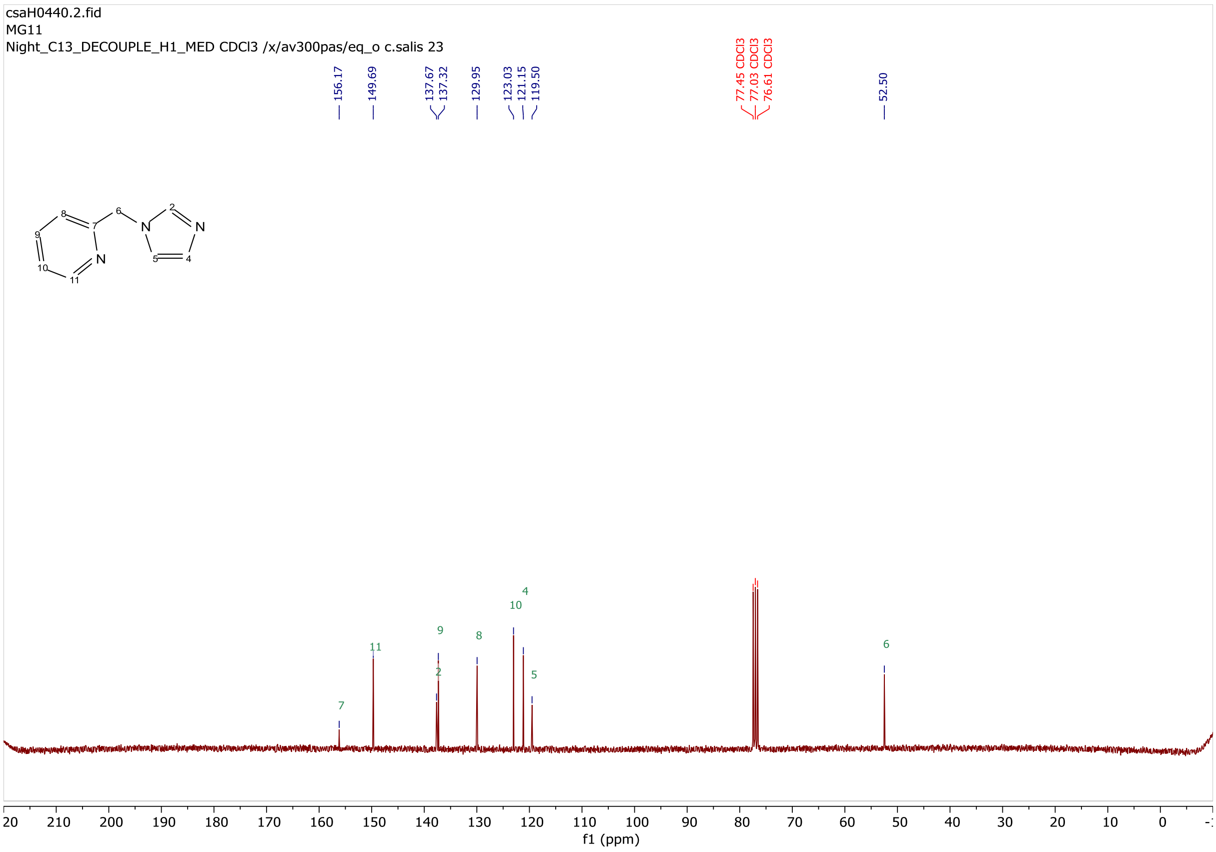

Figure S4: ^{13}C NMR spectrum of **2**.

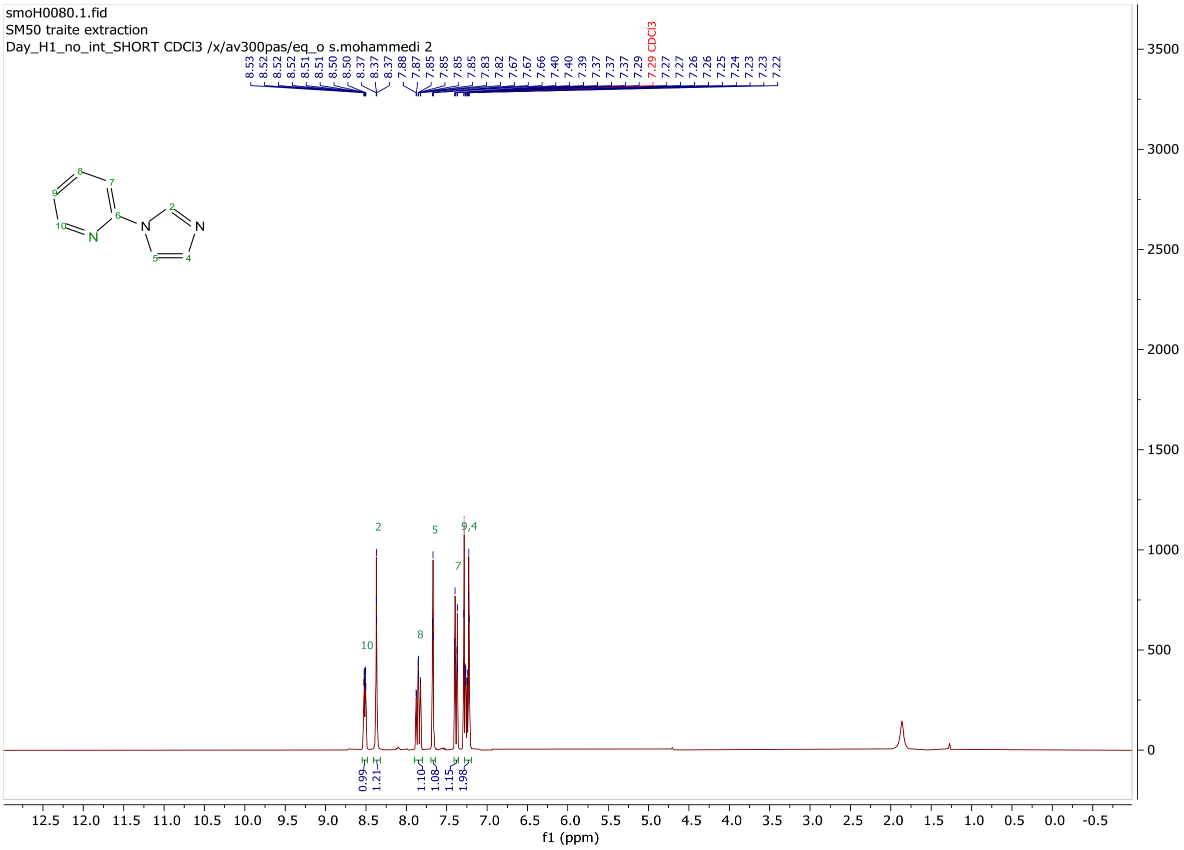

Figure S5: ^{1}H NMR spectrum of **3**.

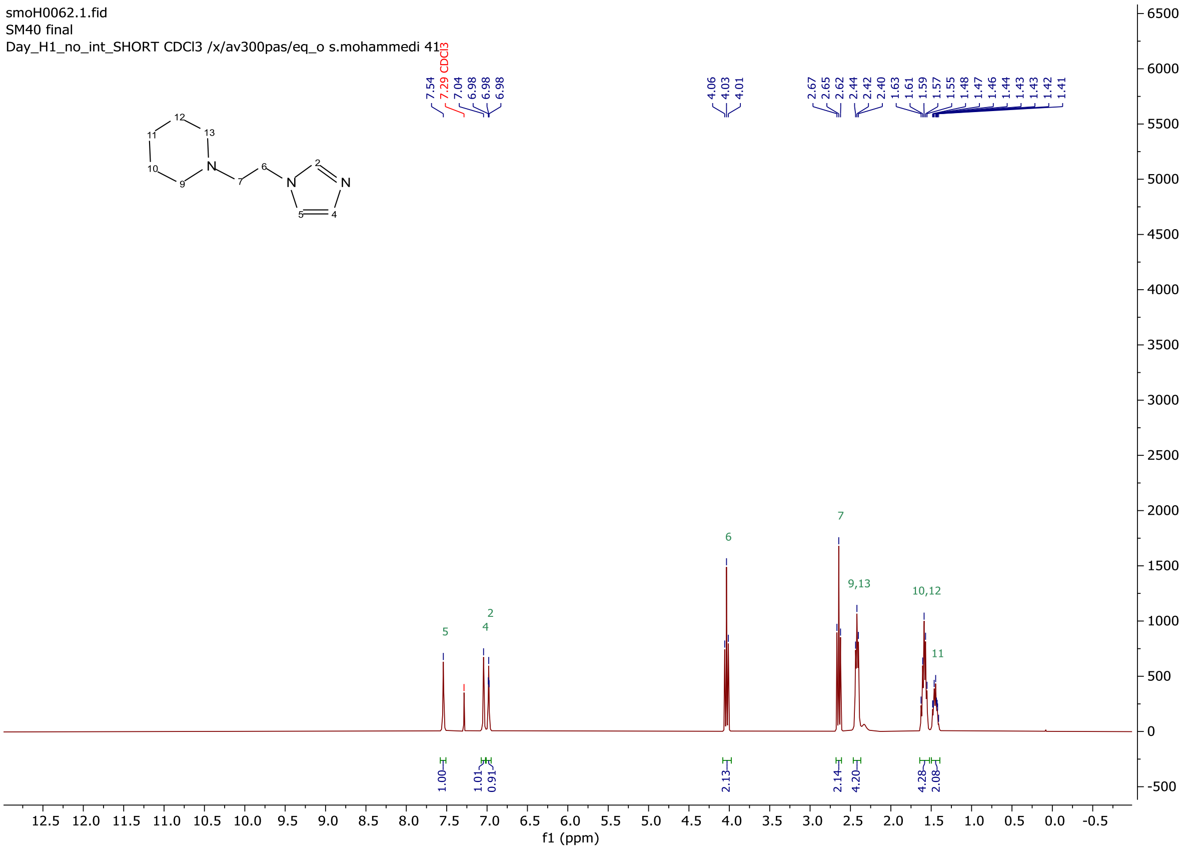

Figure S6: ^{1}H NMR spectrum of **4**.

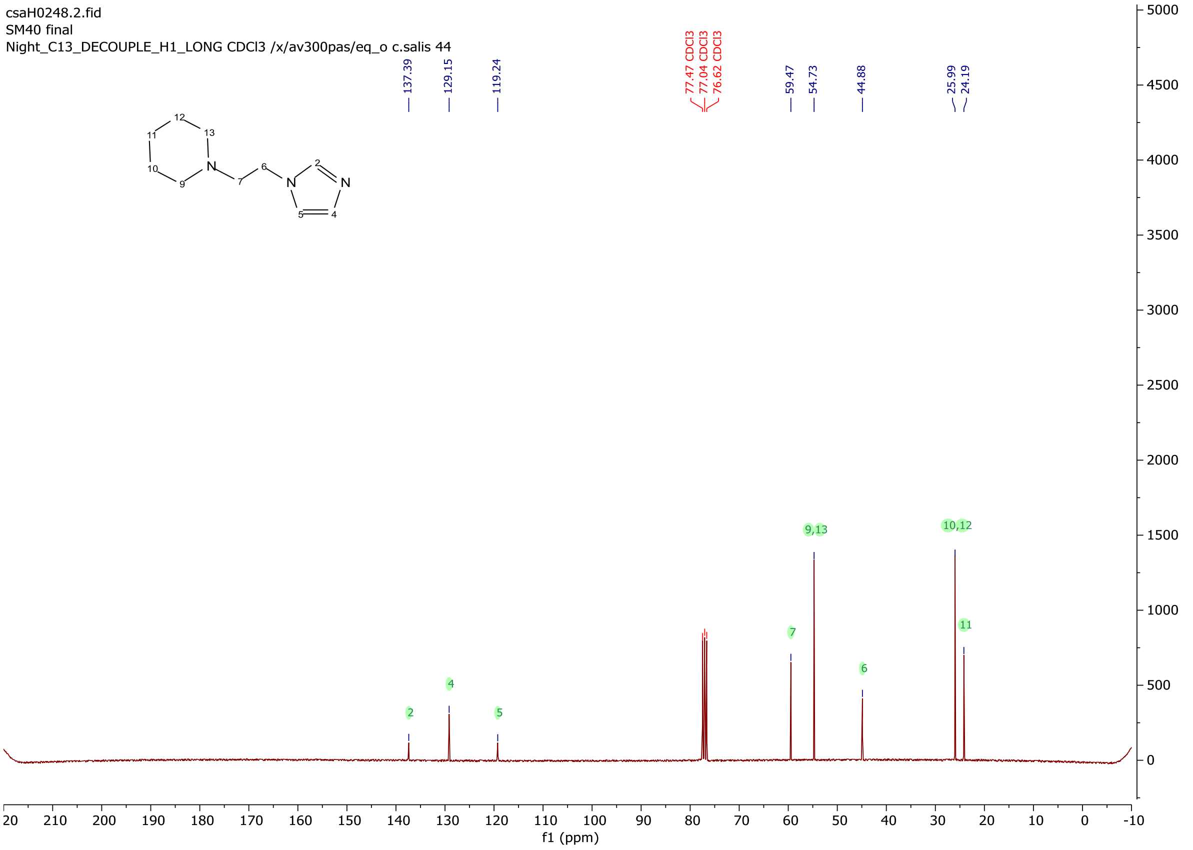

Figure S7: ^{13}C NMR spectrum of **4**.

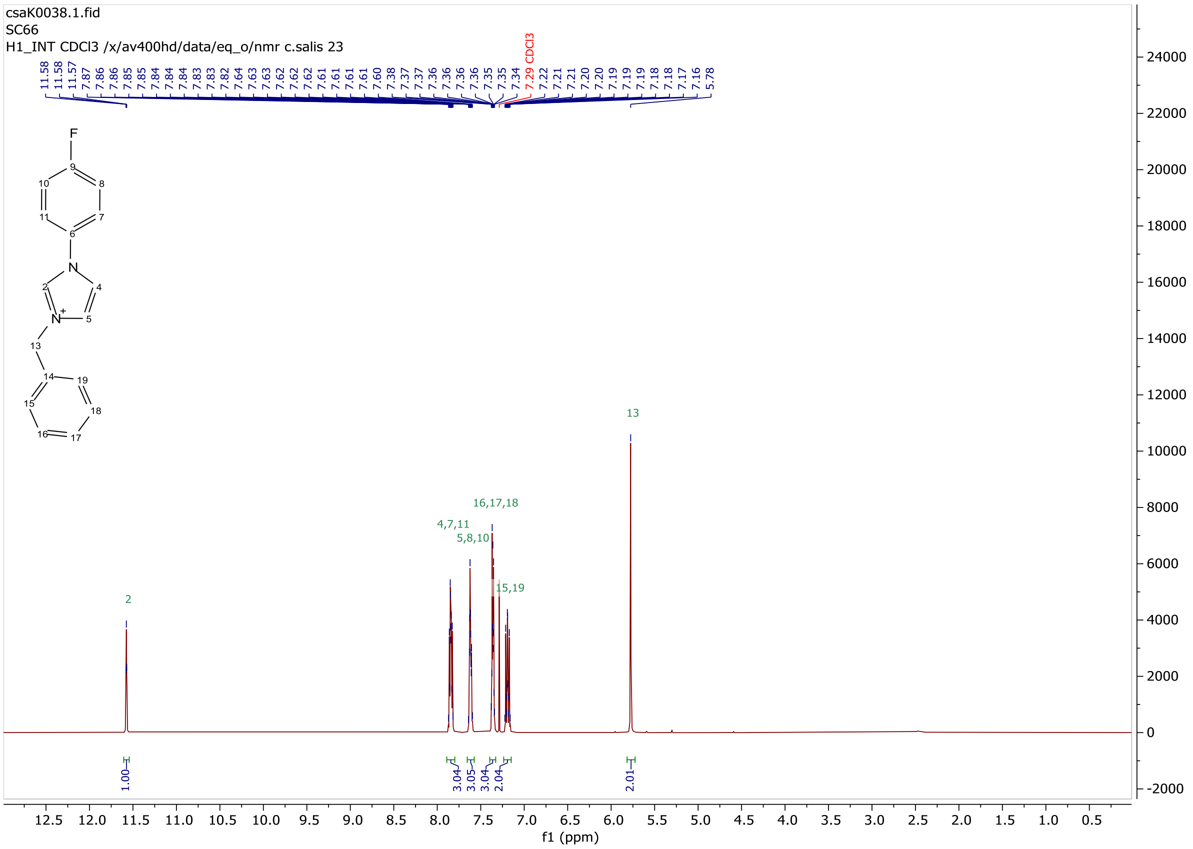

Figure S8: ^{1}H NMR spectrum of **5**.

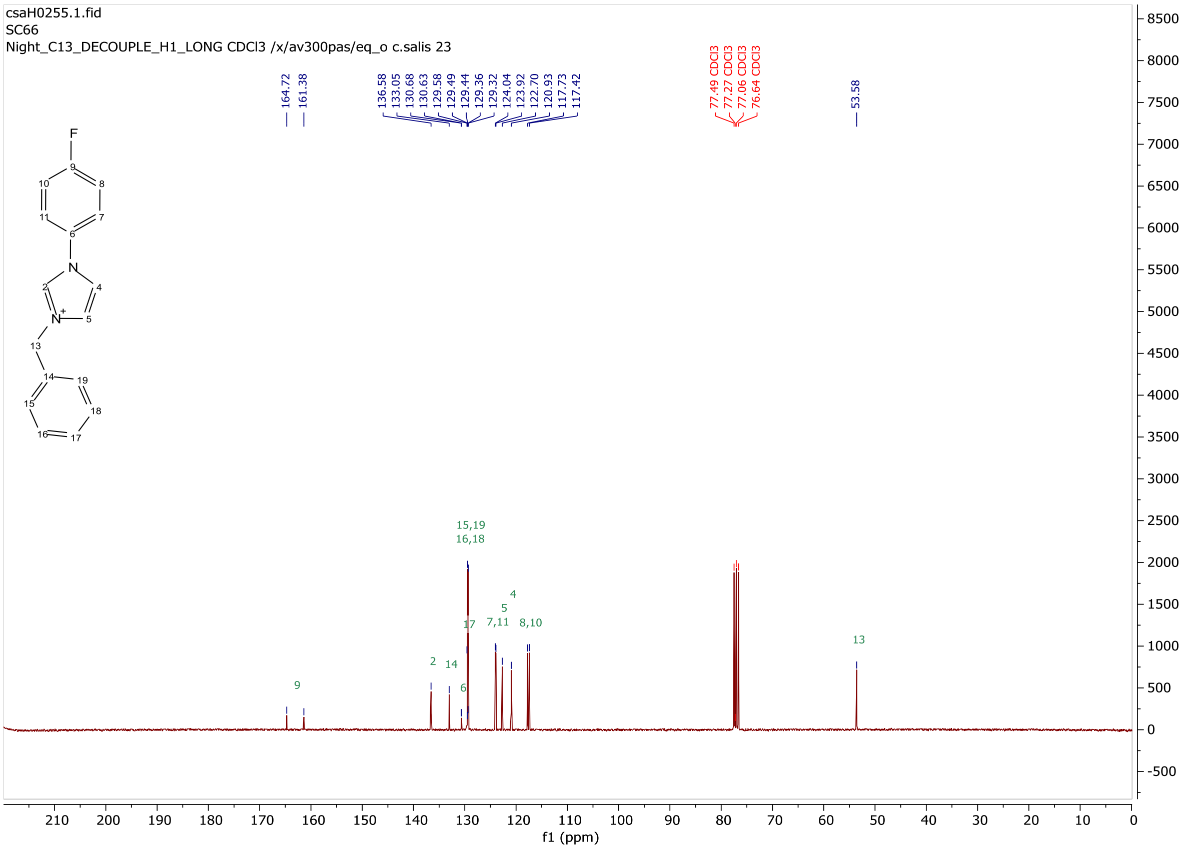

Figure S9: ^{13}C NMR spectrum of **5**.

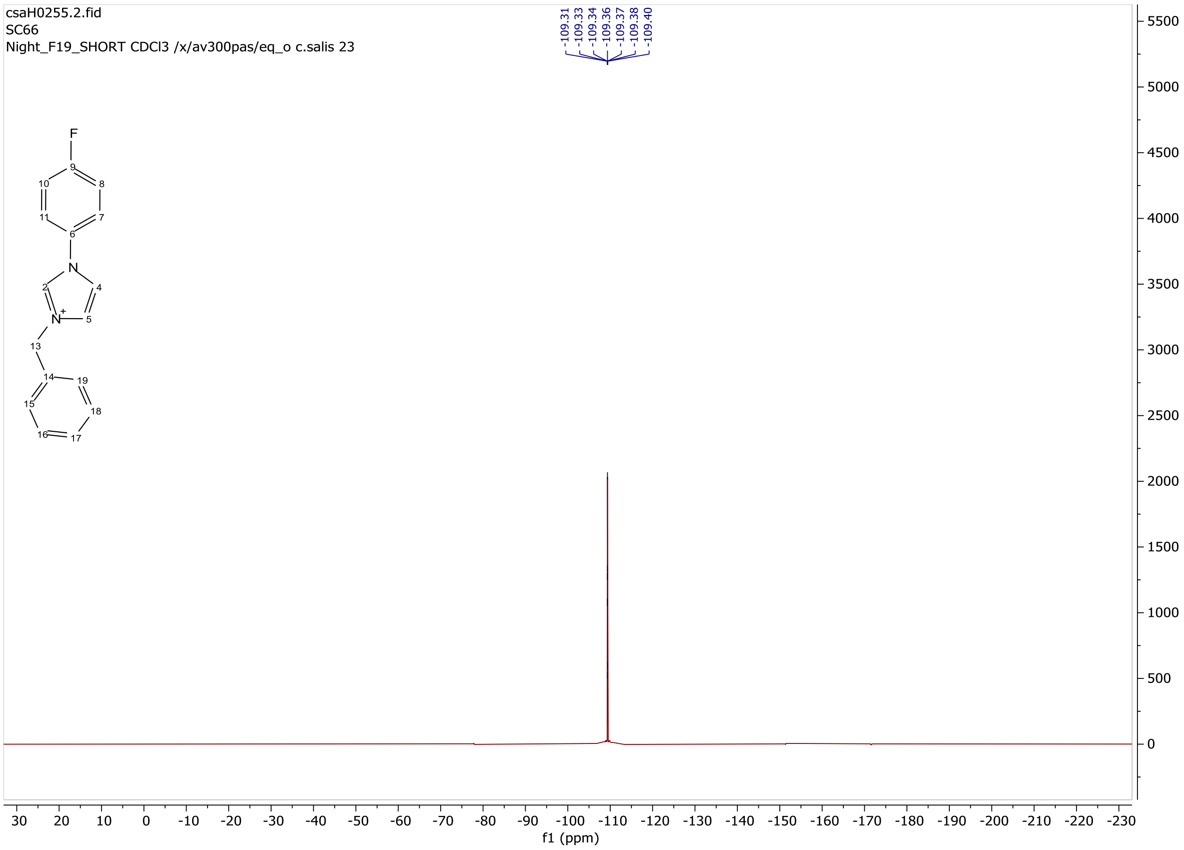

Figure S10: ^{19}F NMR spectrum of **5**.

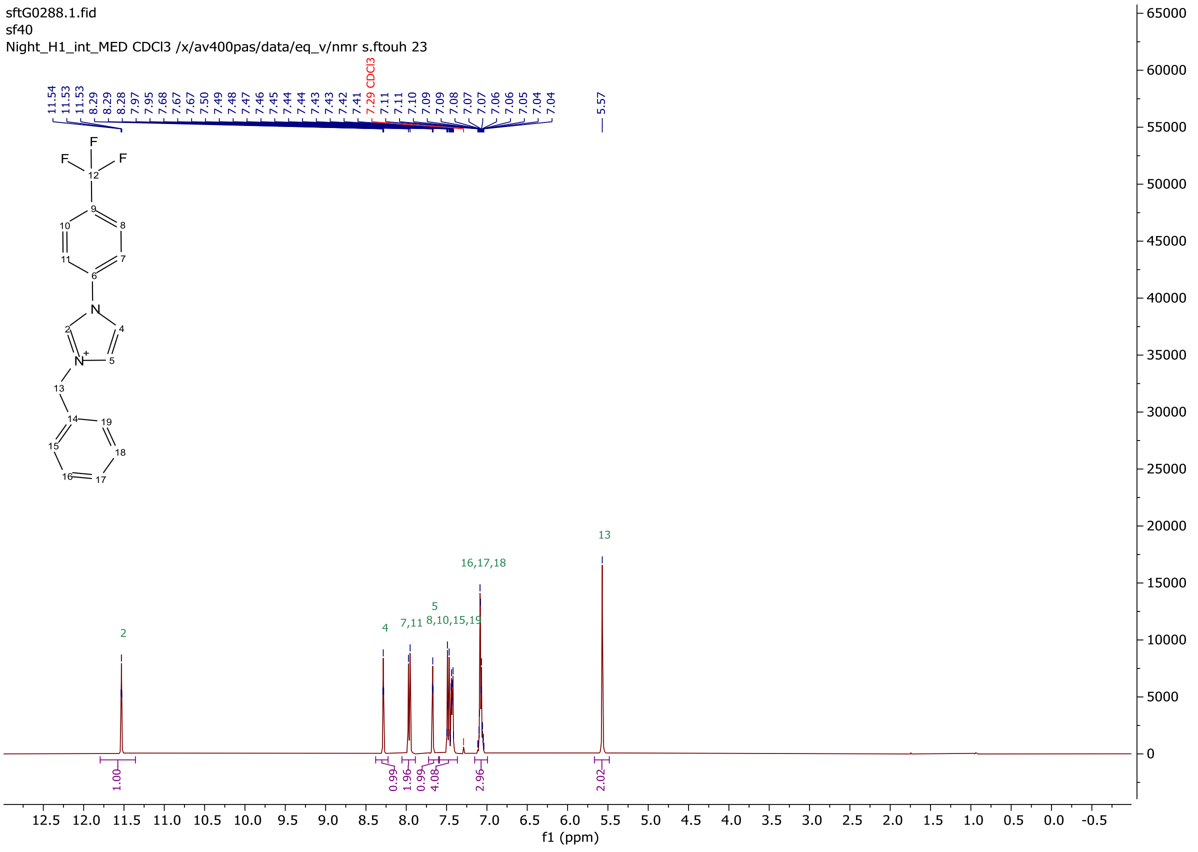

Figure S11: ^{1}H NMR spectrum of **6**.

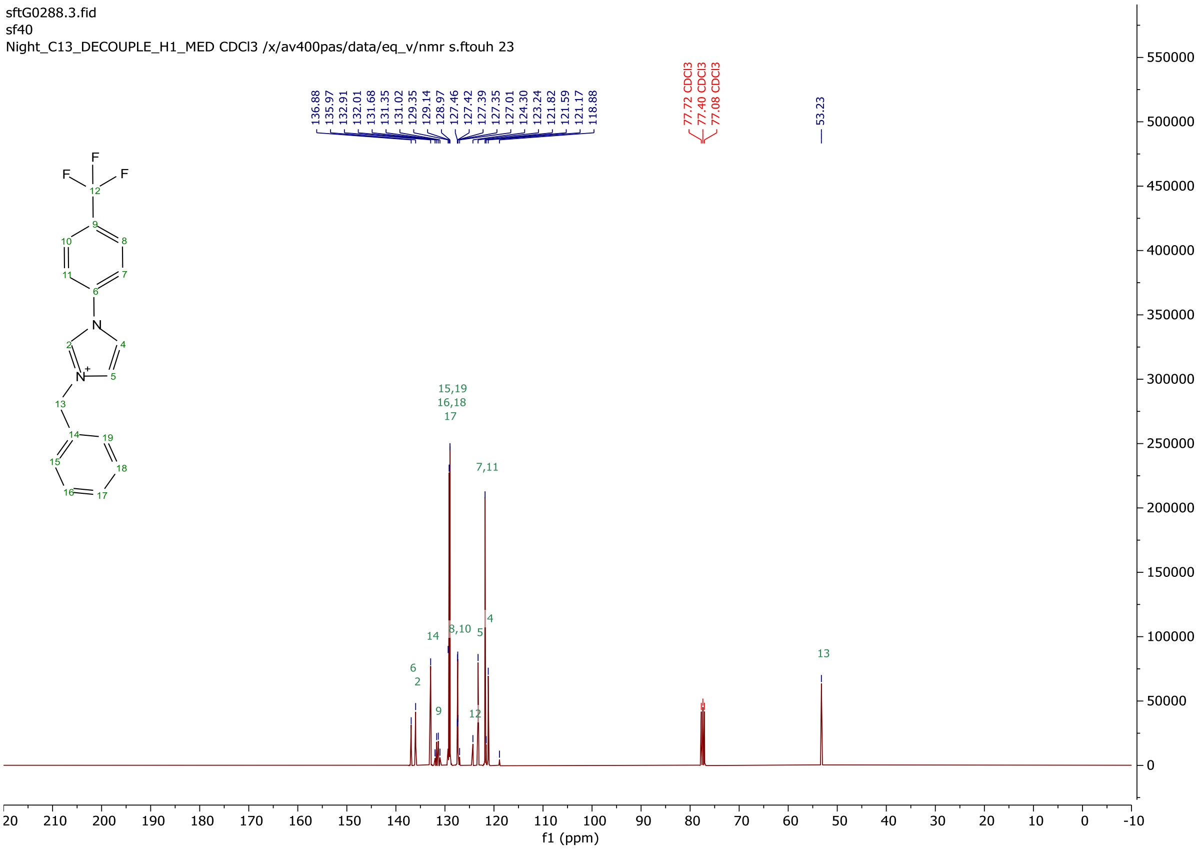

Figure S12: ^{13}C NMR spectrum of **6**.

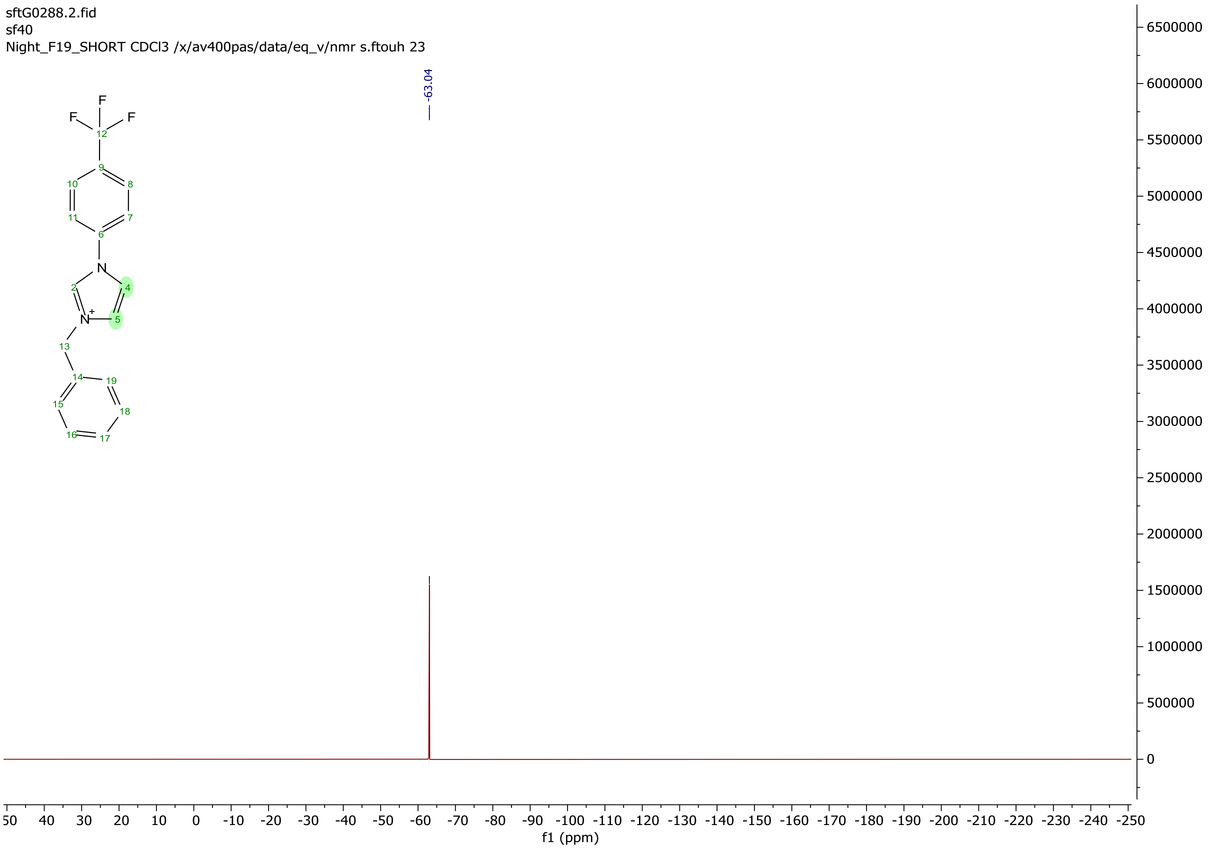

Figure S13: ^{19}F NMR spectrum of **6**.

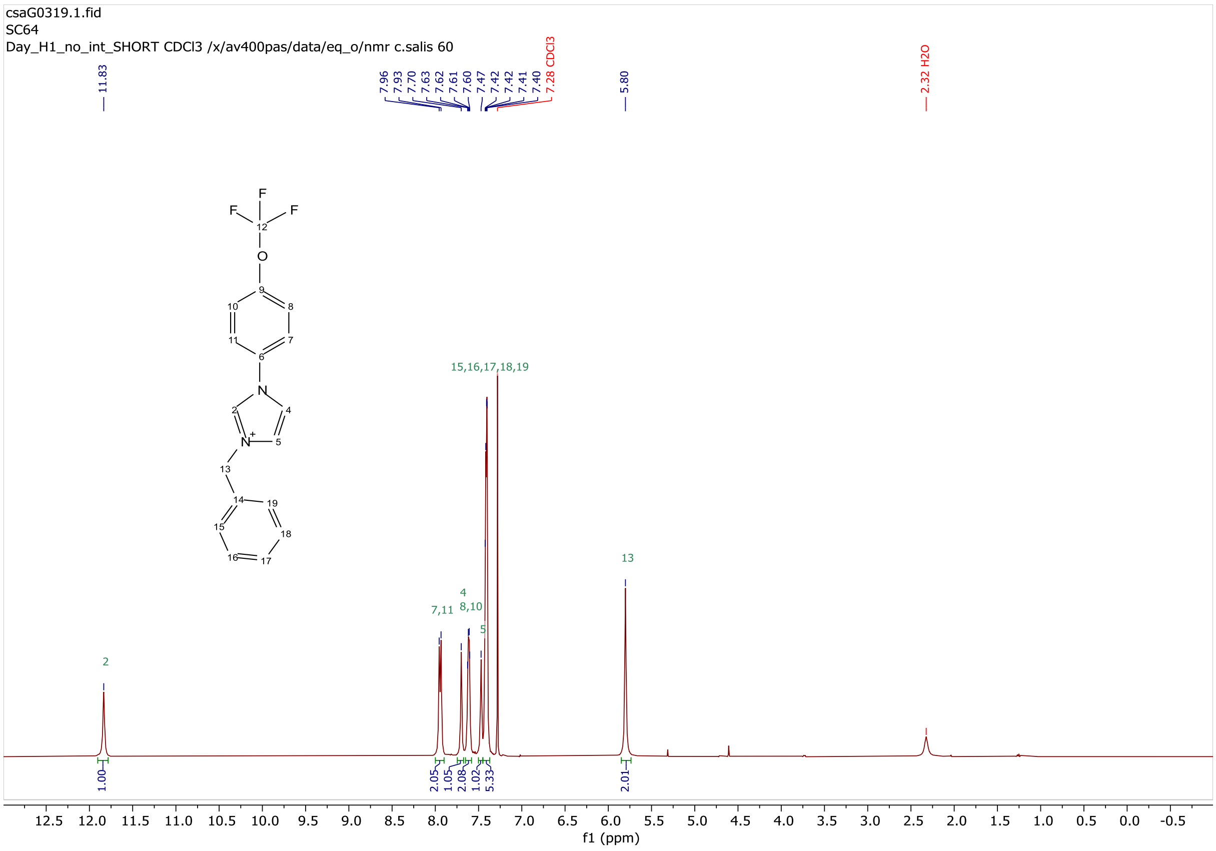

Figure S14: ^{1}H NMR spectrum of **7**.

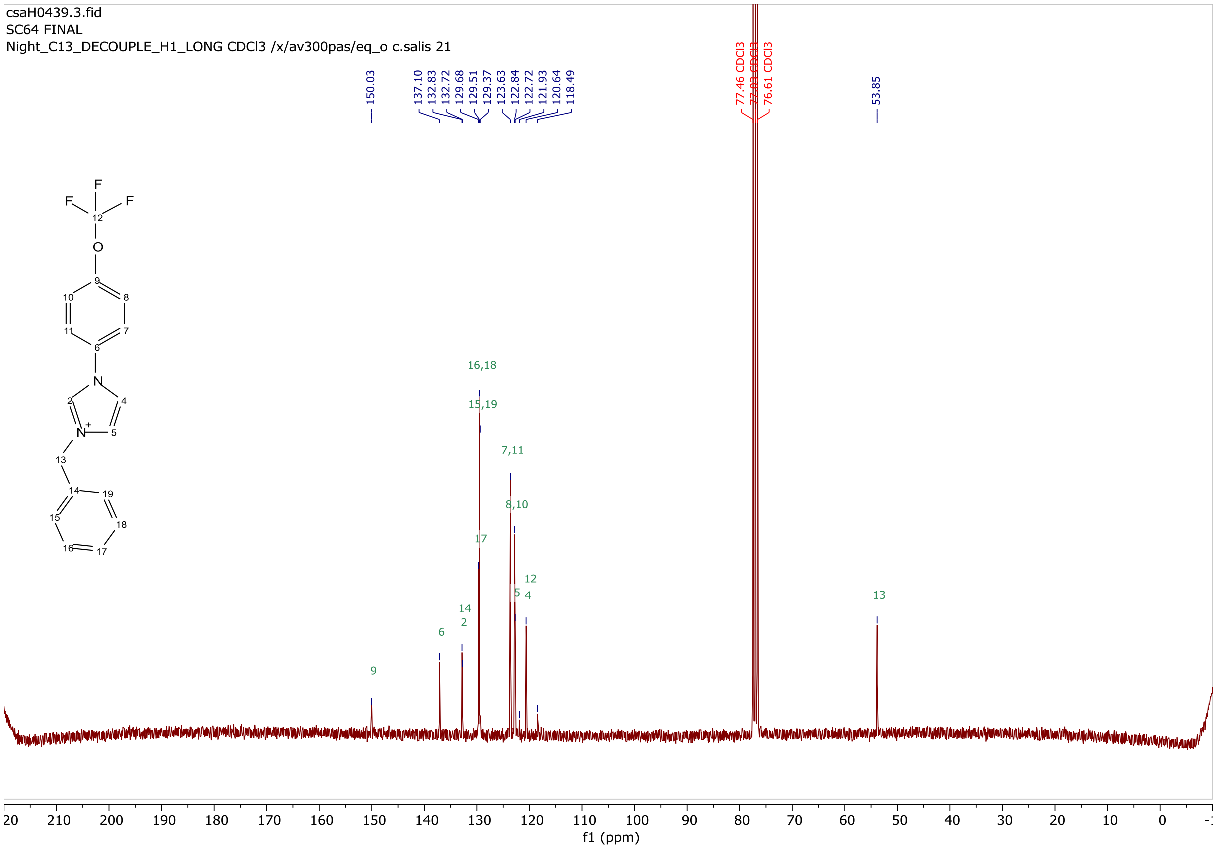

Figure S15: ^{13}C NMR spectrum of **7**.

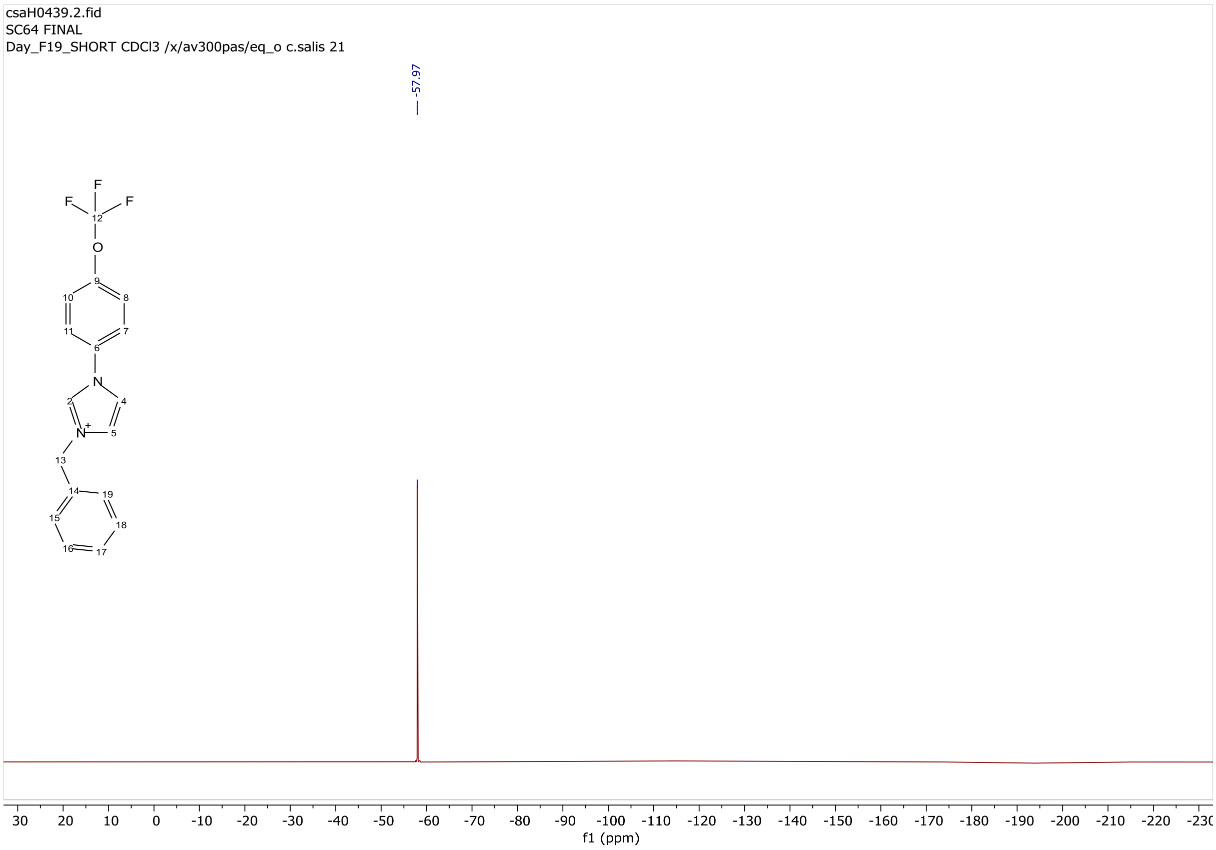

Figure S16: ^{19}F NMR spectrum of **7**.

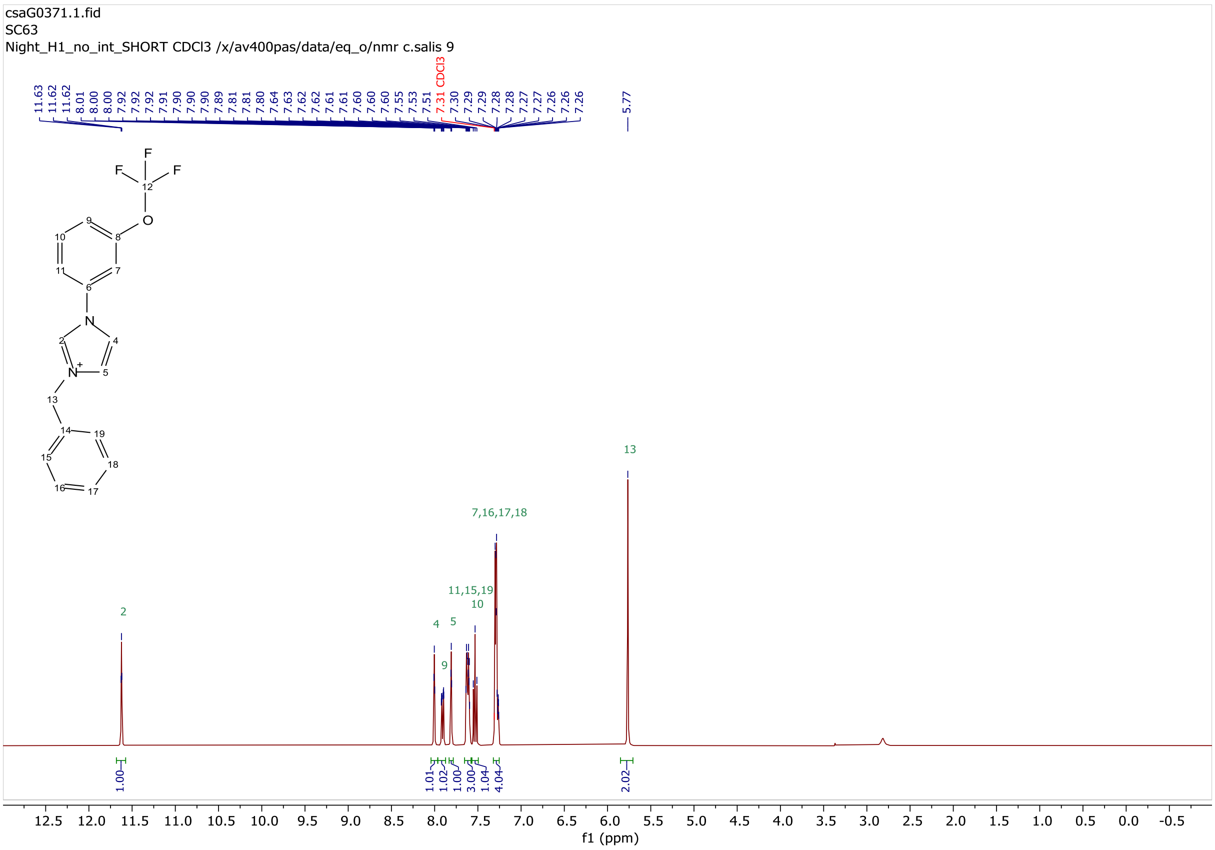

Figure S17: ^{1}H NMR spectrum of **8**.

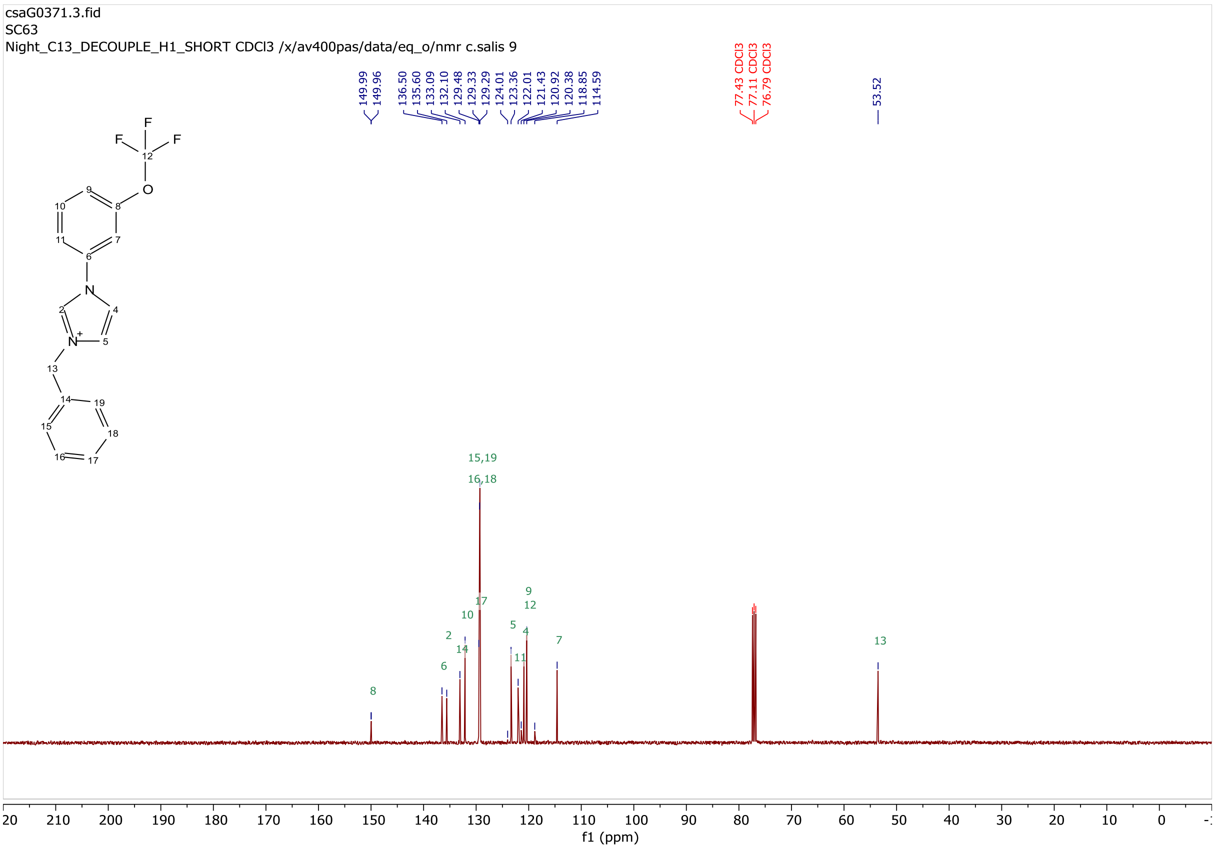

Figure S18: ^{13}C NMR spectrum of **8**.

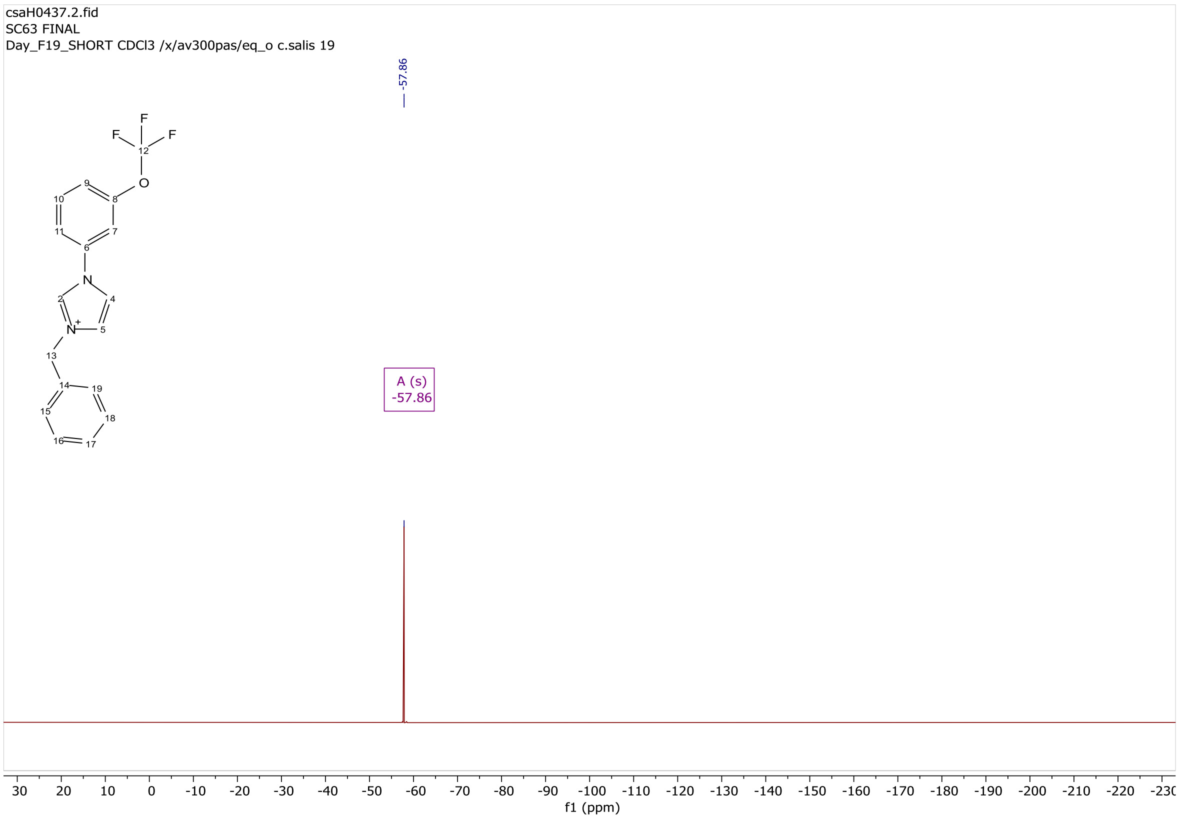

Figure S19: ^{19}F NMR spectrum of **8**.

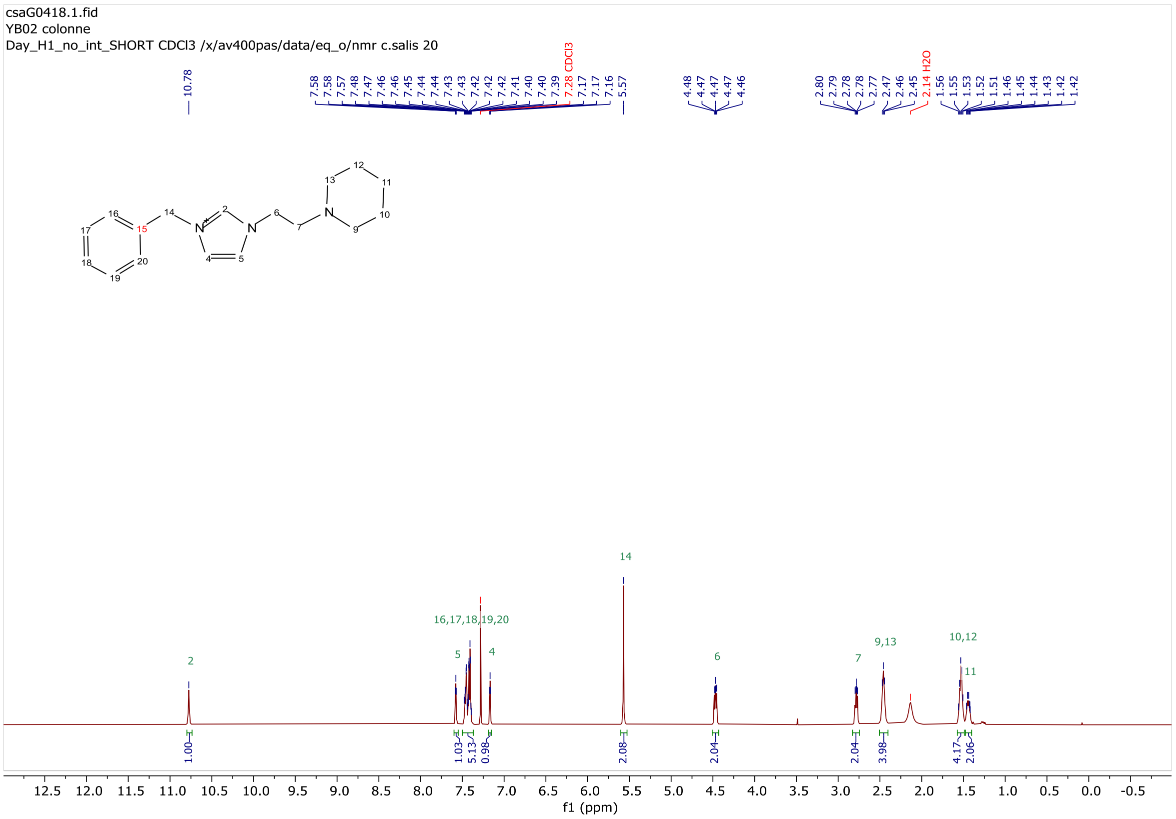

Figure S20: ^{1}H NMR spectrum of **10**.

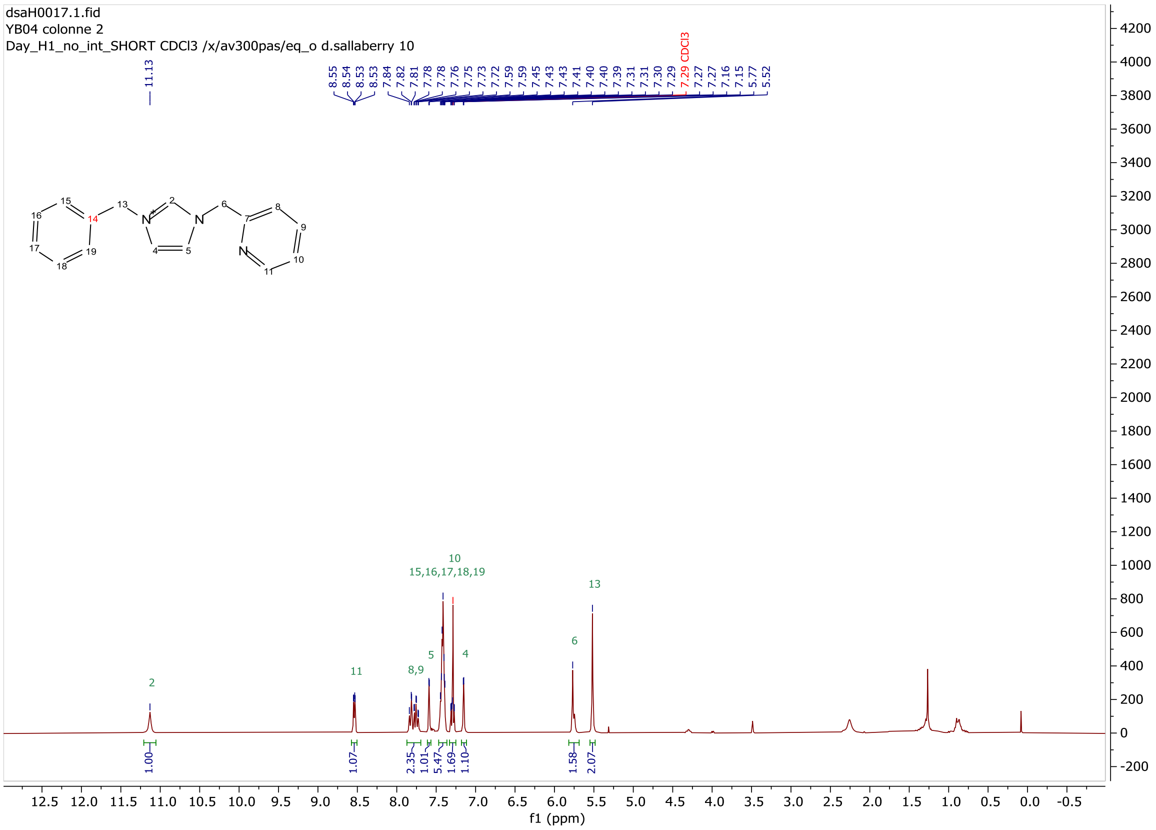

Figure S21: ^{1}H NMR spectrum of **11**.

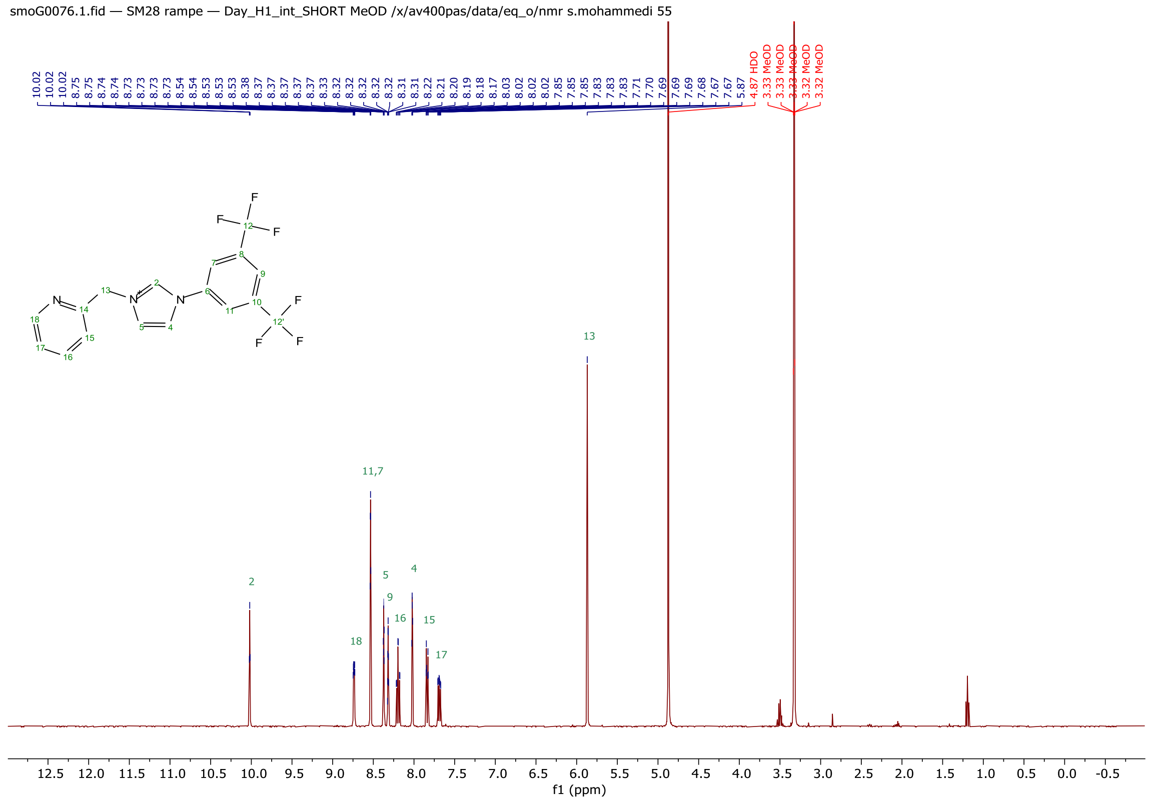

Figure S22: ^{1}H NMR spectrum of **12**.

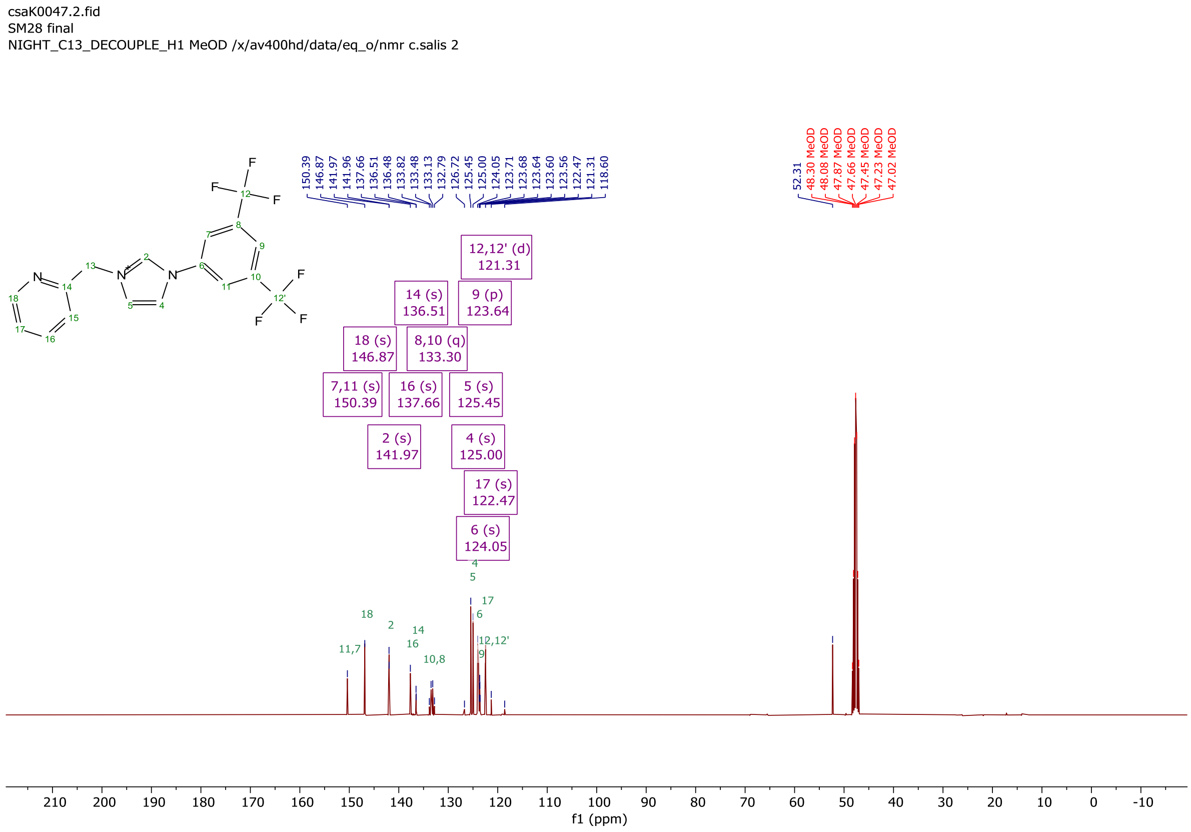

Figure S23: ^{13}C NMR spectrum of **12**.

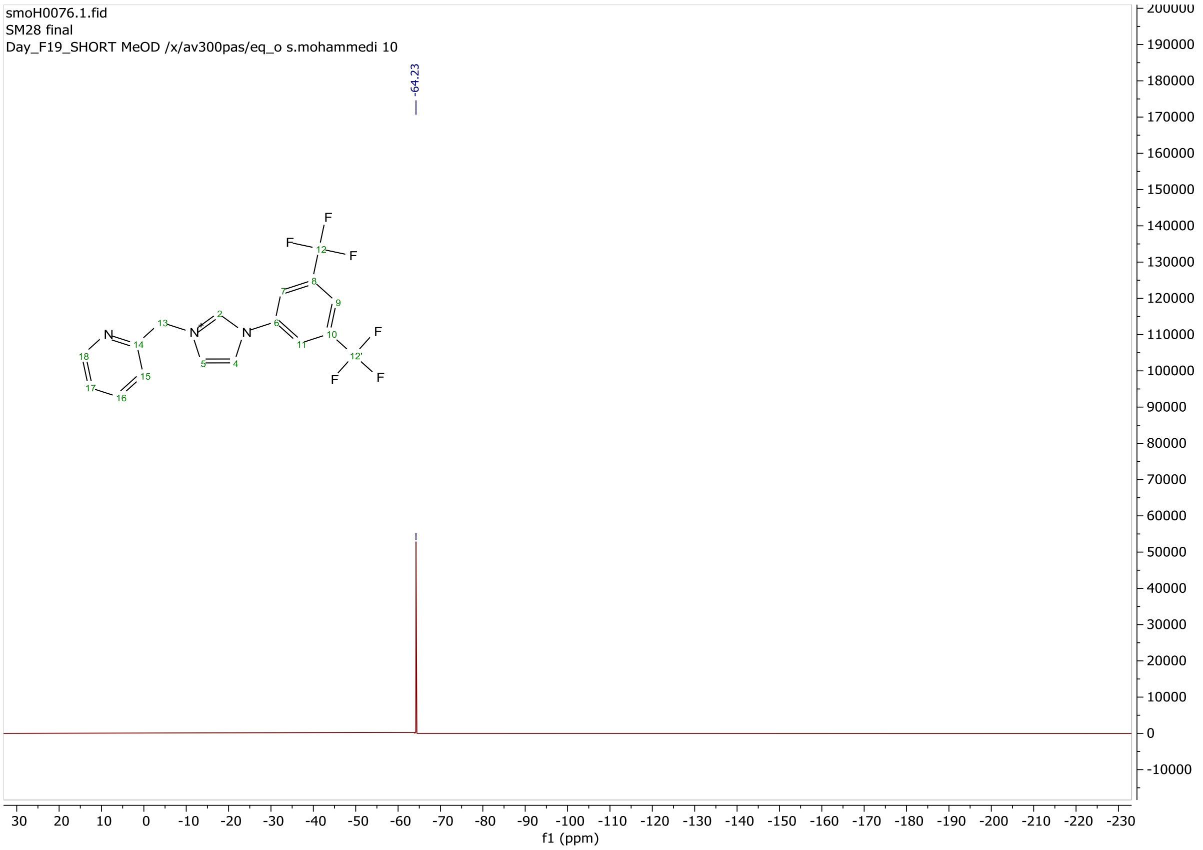

Figure S24: ^{19}F NMR spectrum of **12**.

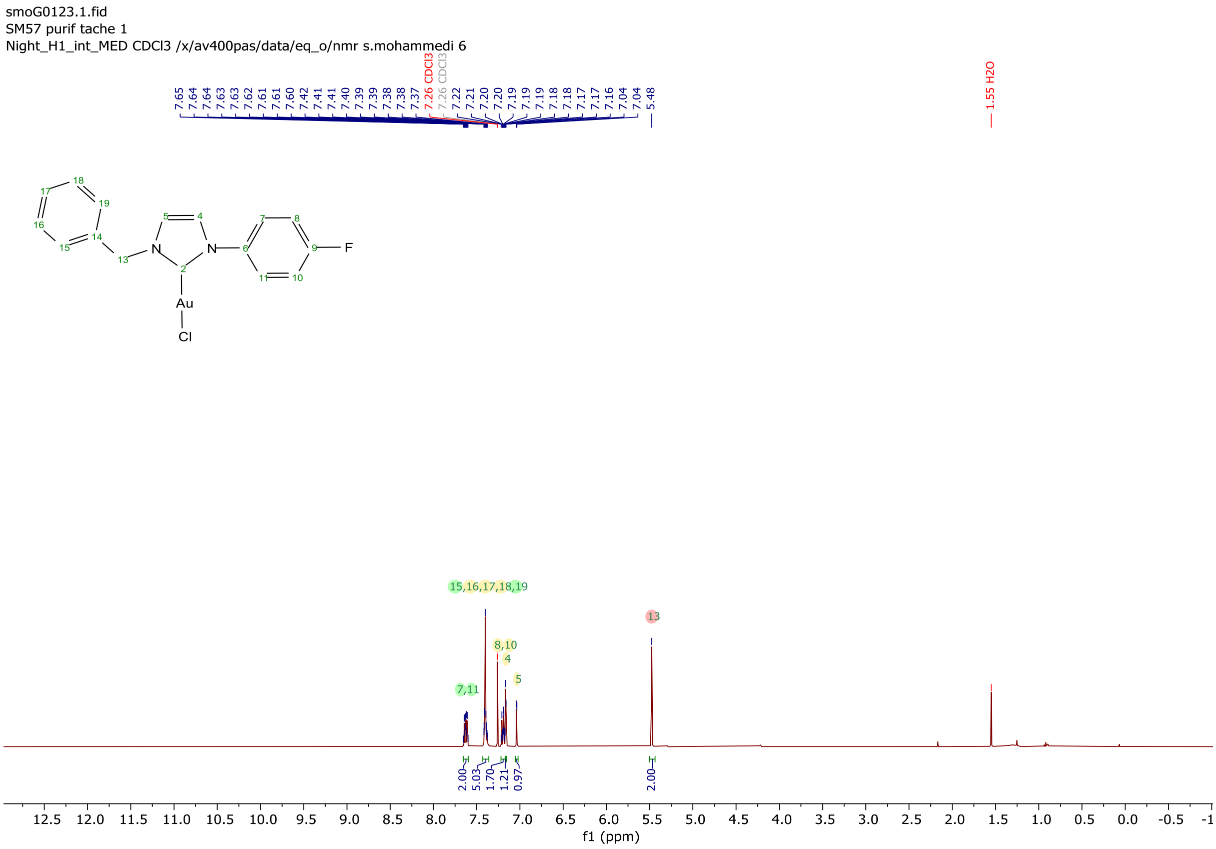

Figure S25: ^{1}H NMR spectrum of **13**.

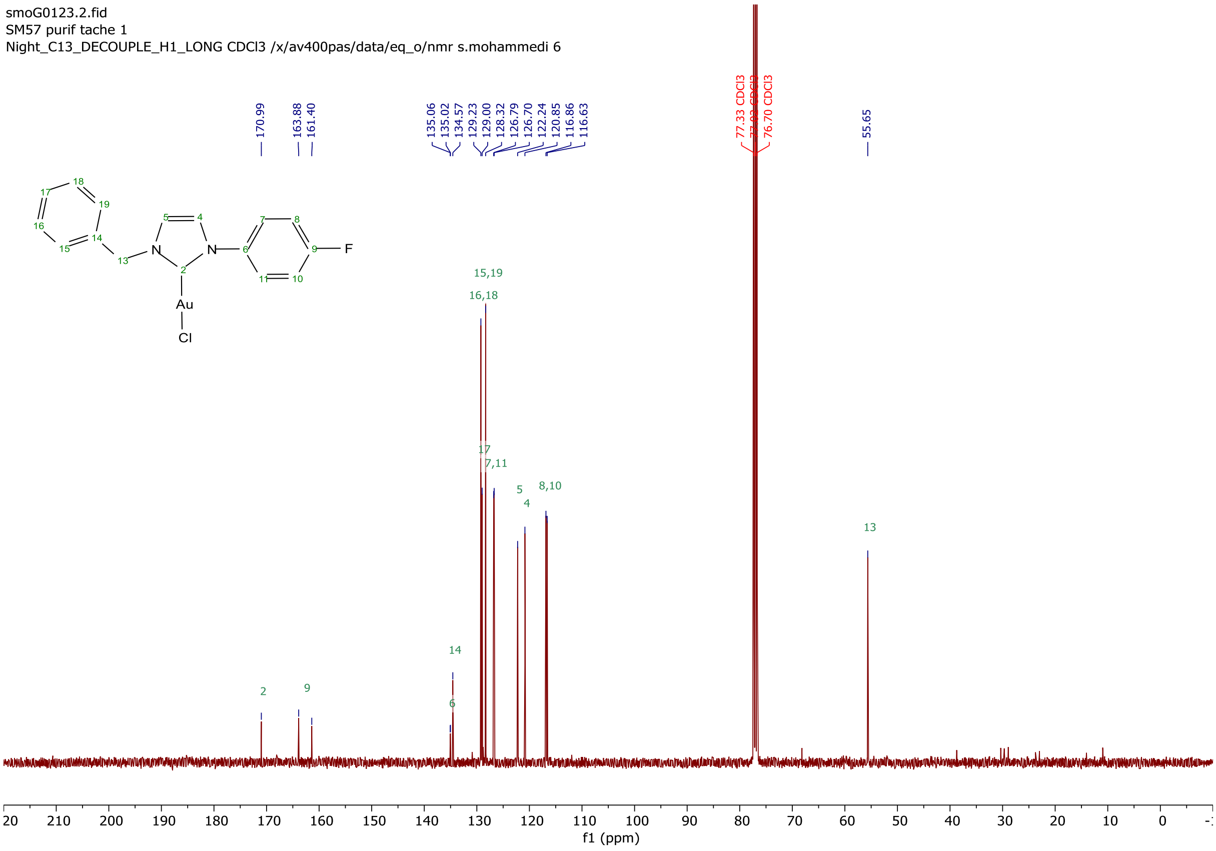

Figure S26: ^{13}C NMR spectrum of **13**.

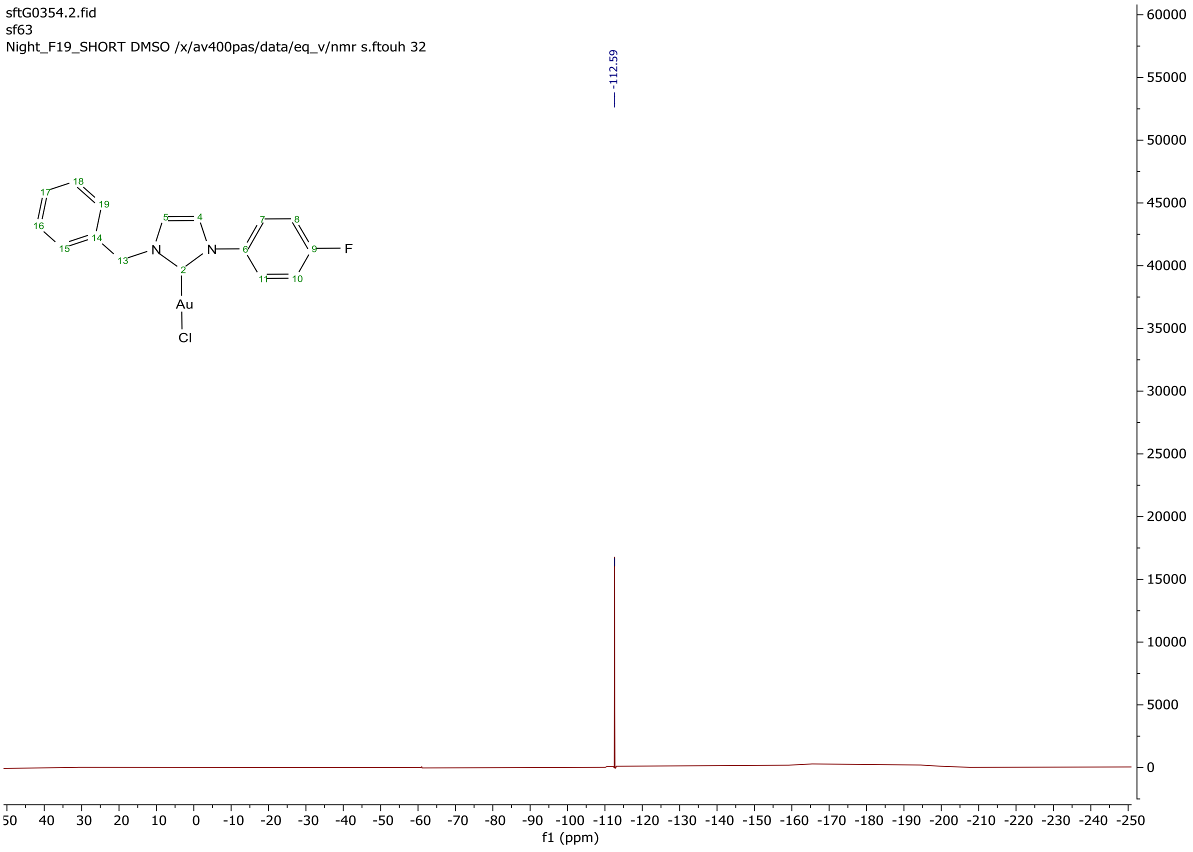

Figure S27: ^{19}F NMR spectrum of **13**.

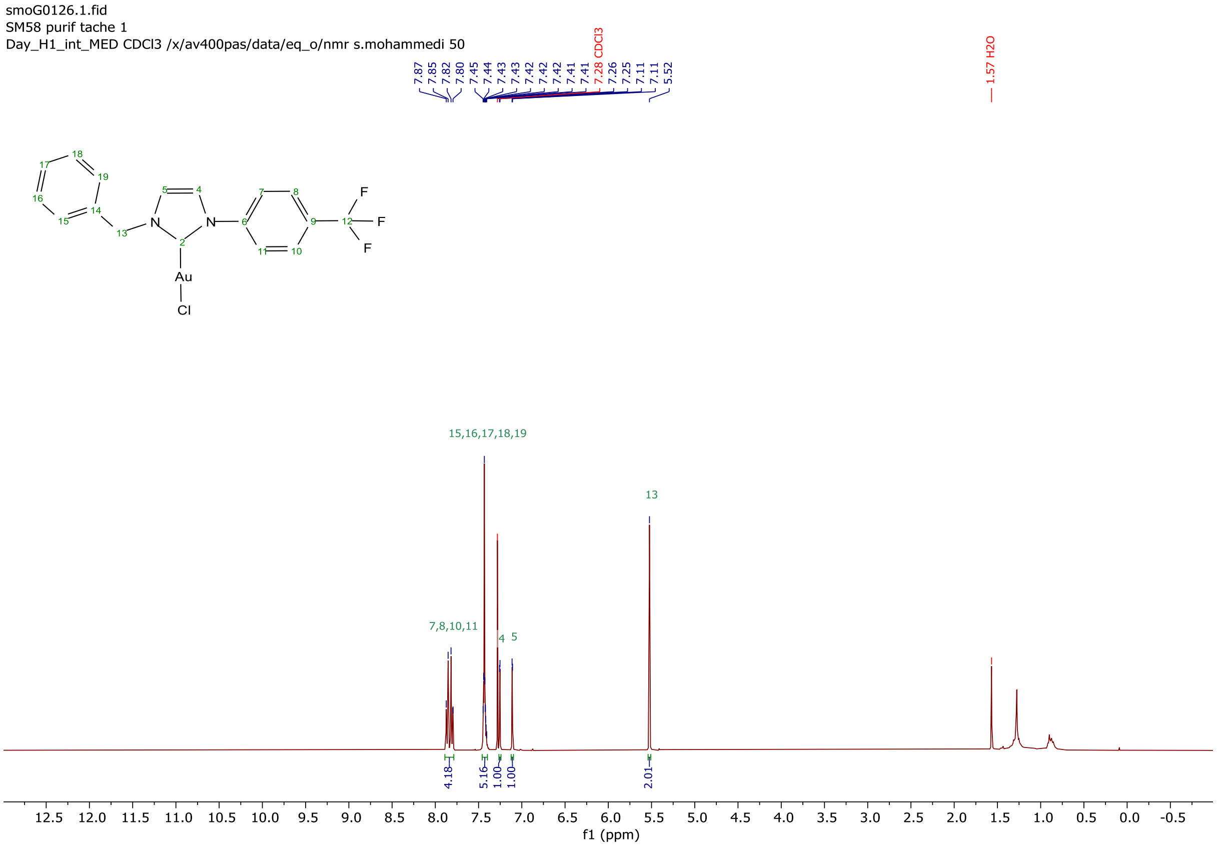

Figure S28: ^{1}H NMR spectrum of **14**.

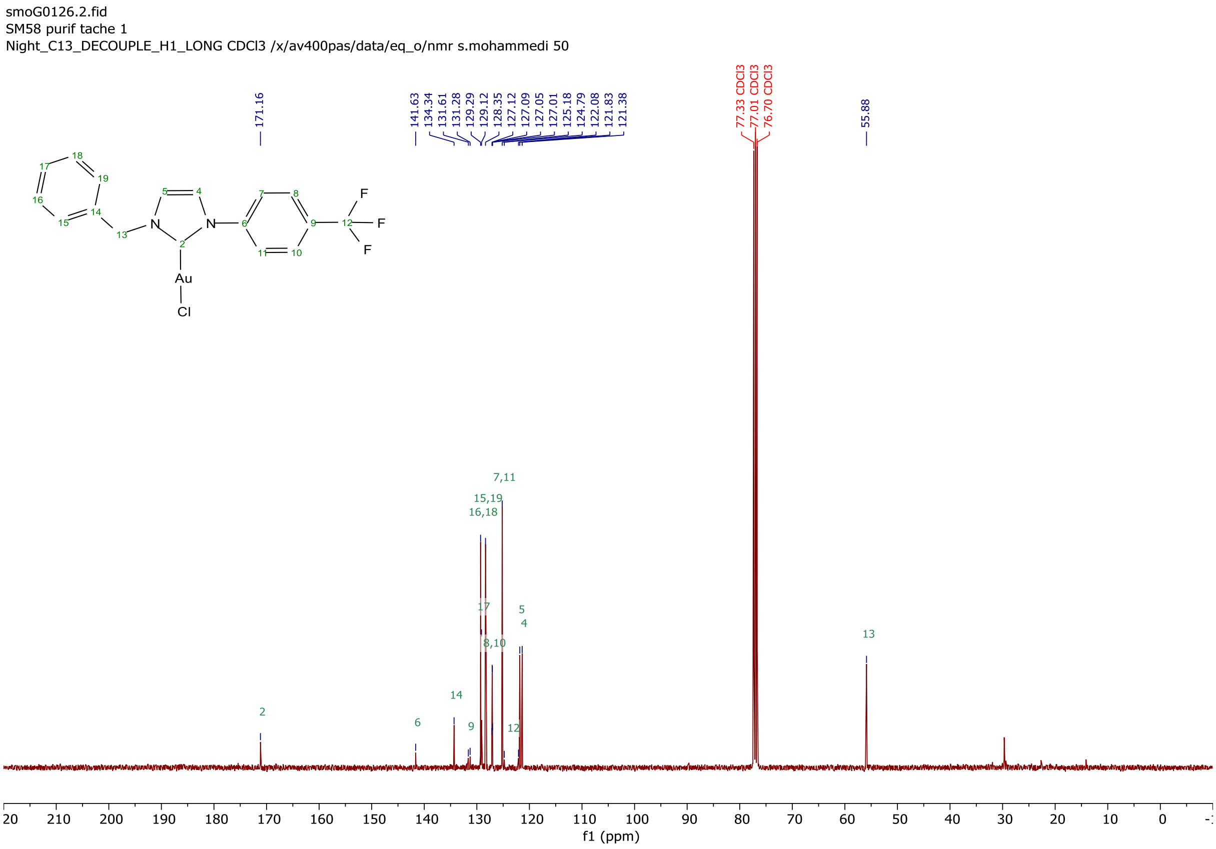

Figure S29: ^{13}C NMR spectrum of **14**.

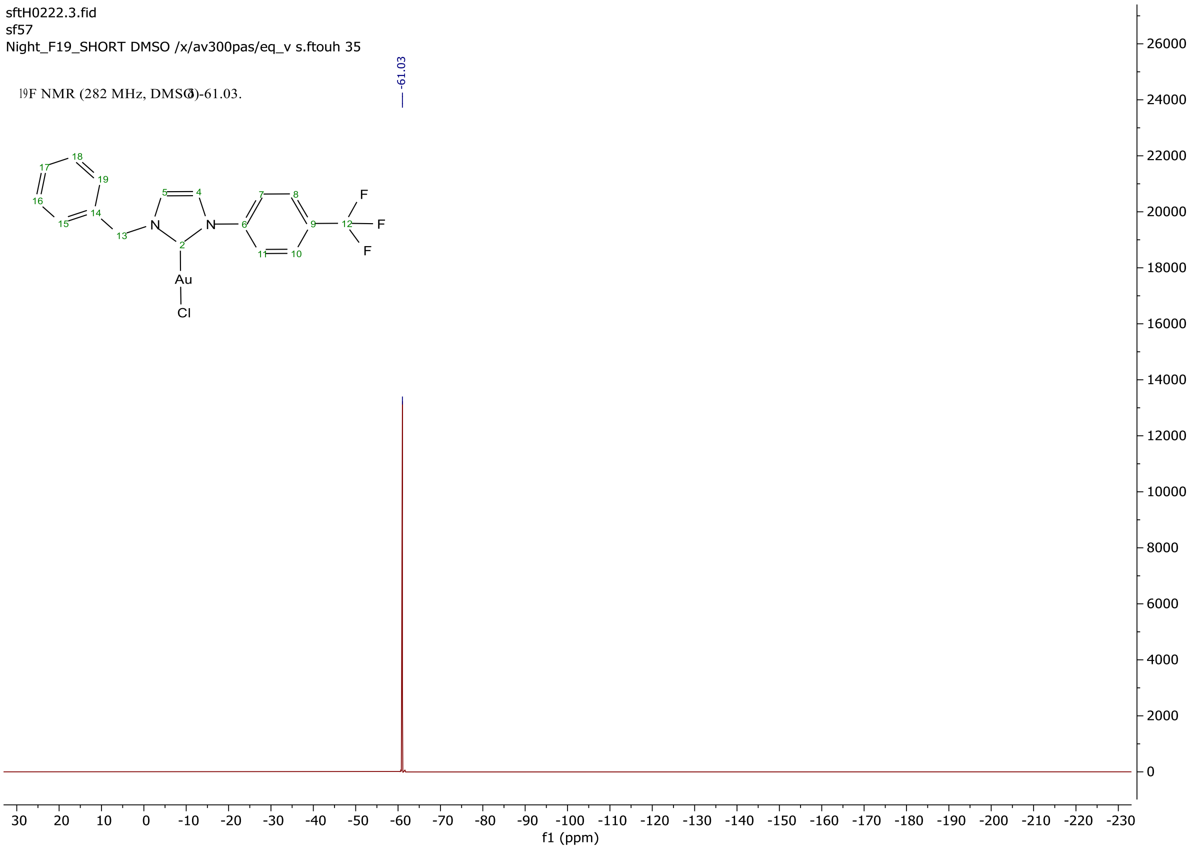

Figure S30: ^{19}F NMR spectrum of **14**.

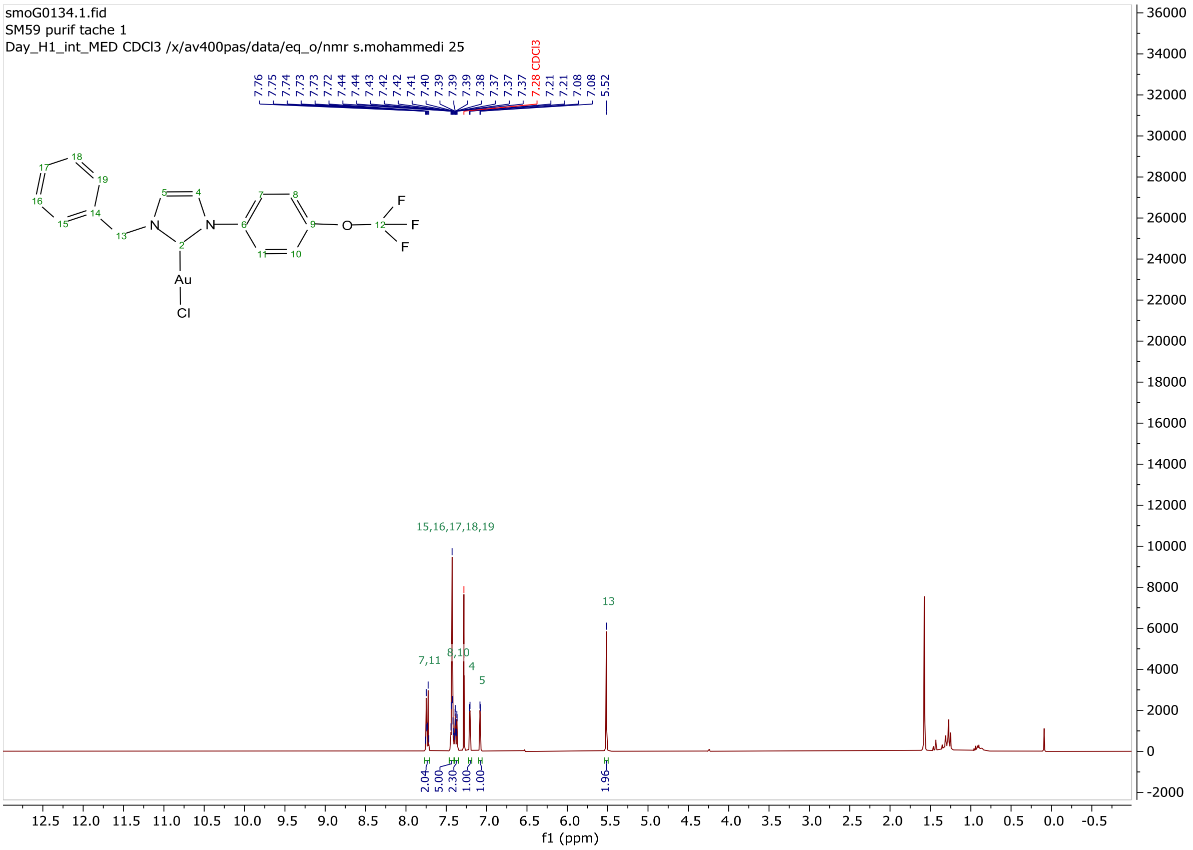

Figure S31: ^{1}H NMR spectrum of **15**.

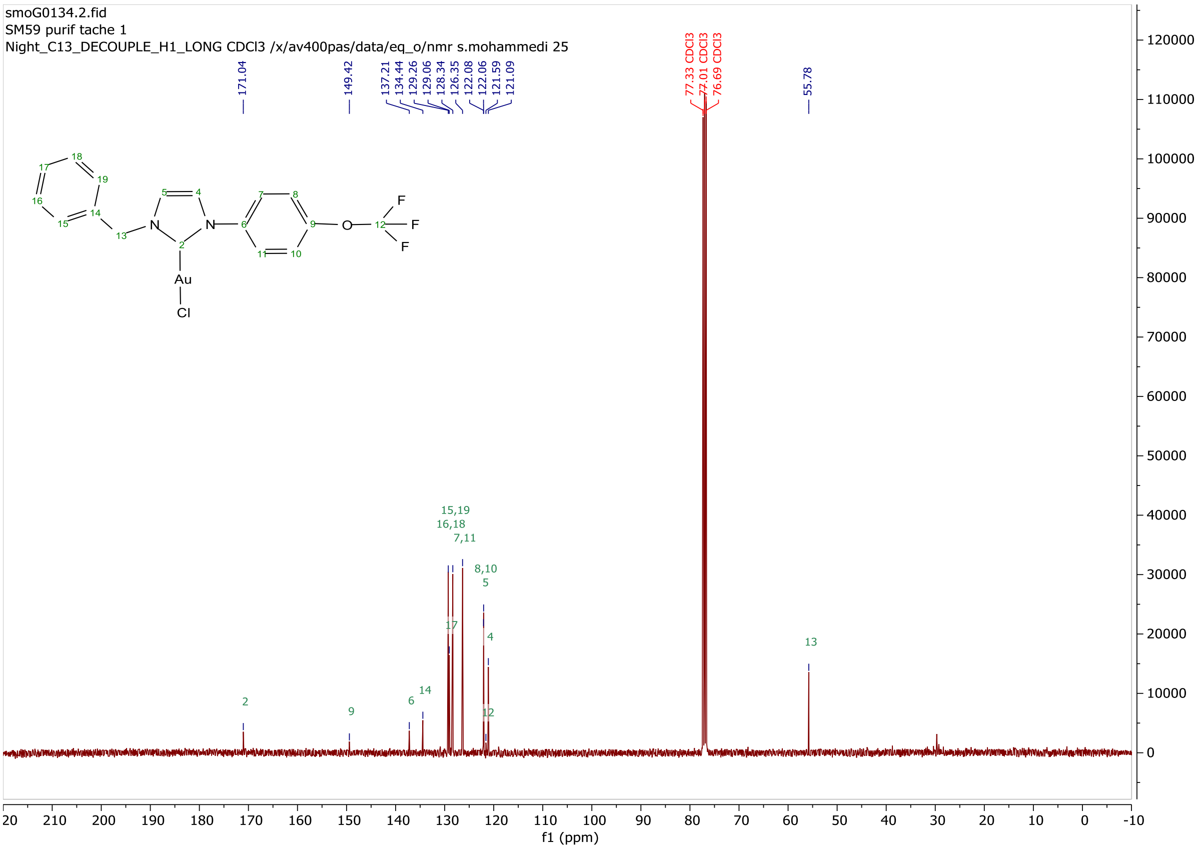

Figure S32: ^{13}C NMR spectrum of **15**.

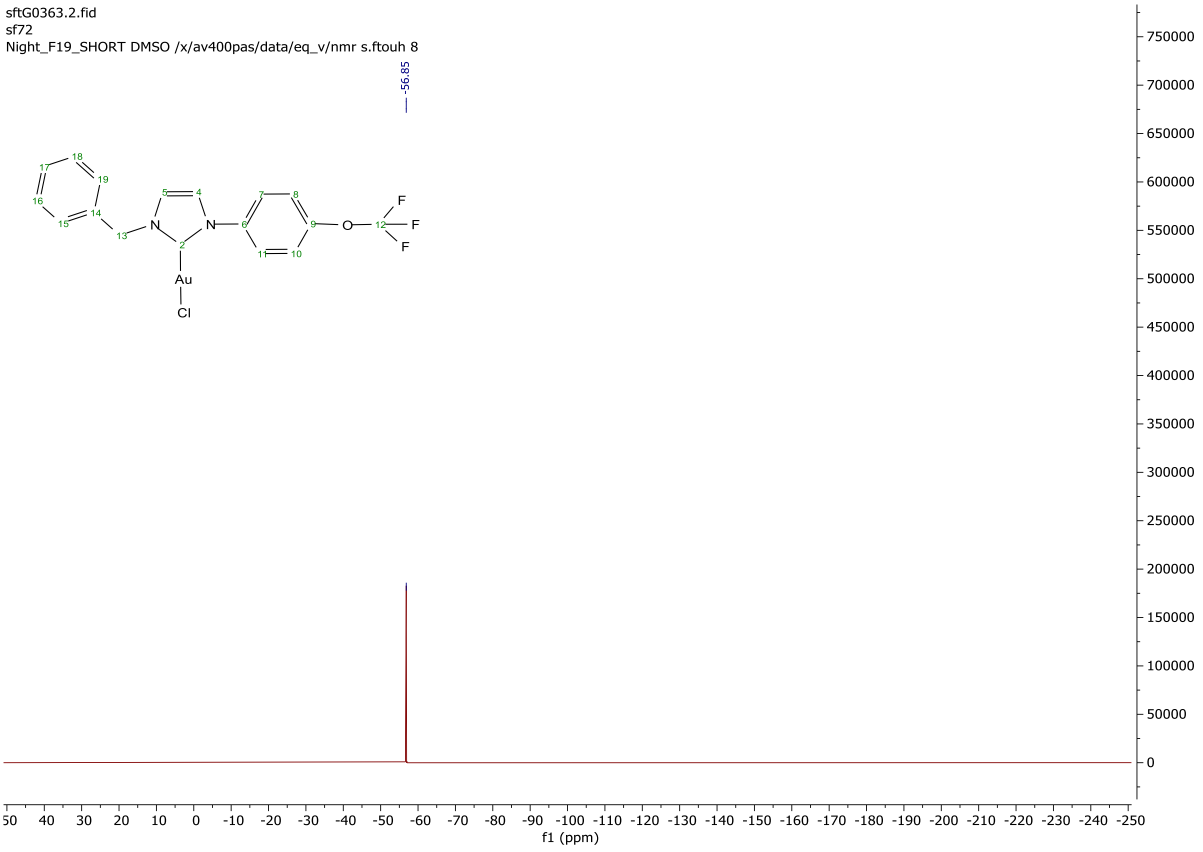

Figure S33: ^{19}F NMR spectrum of **15**.

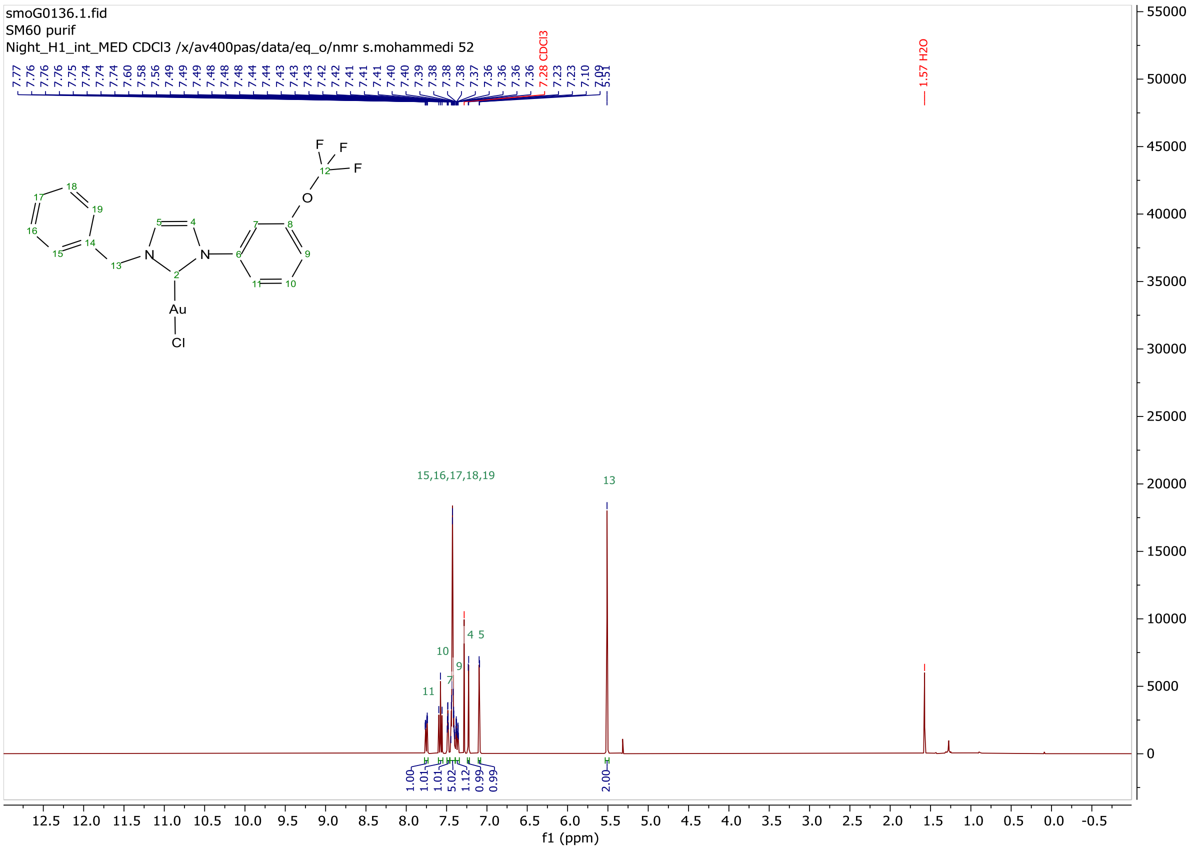

Figure S34: ^{1}H NMR spectrum of **16**.

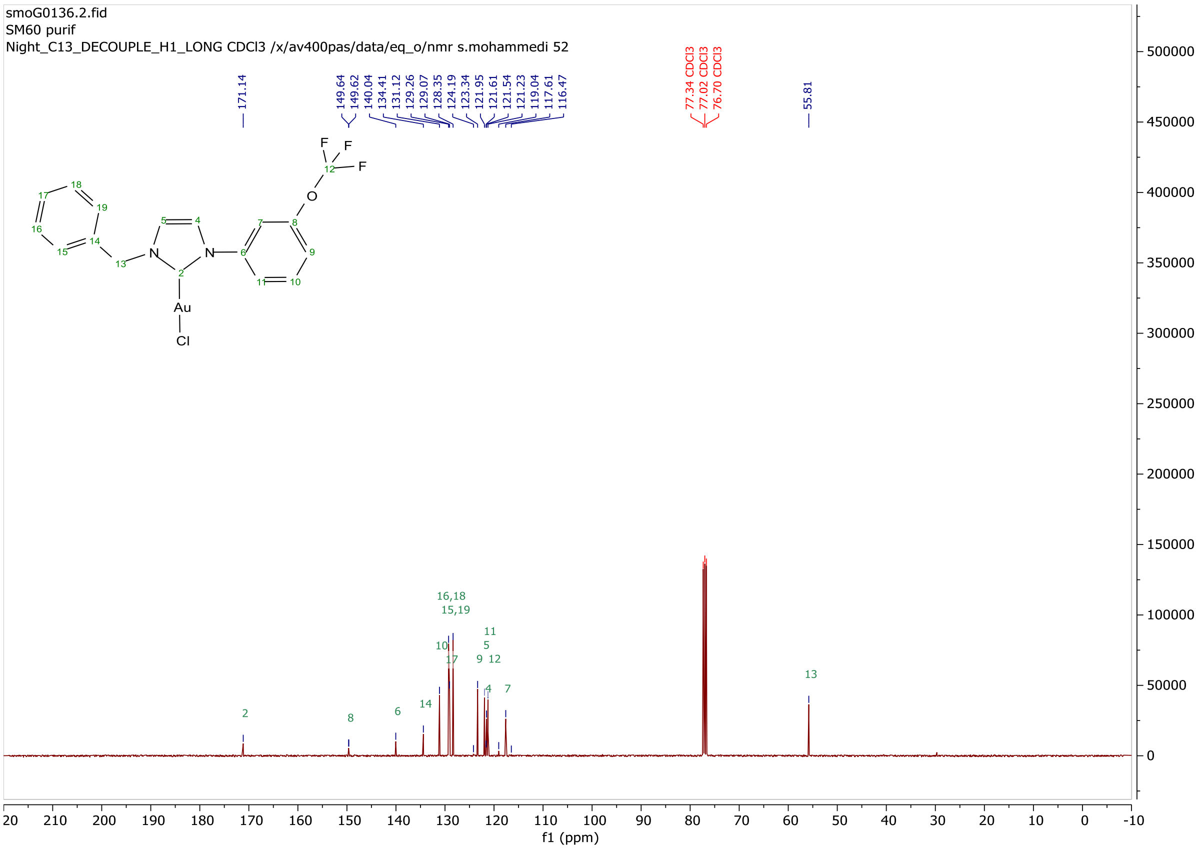

Figure S35: ^{13}C NMR spectrum of **16**.

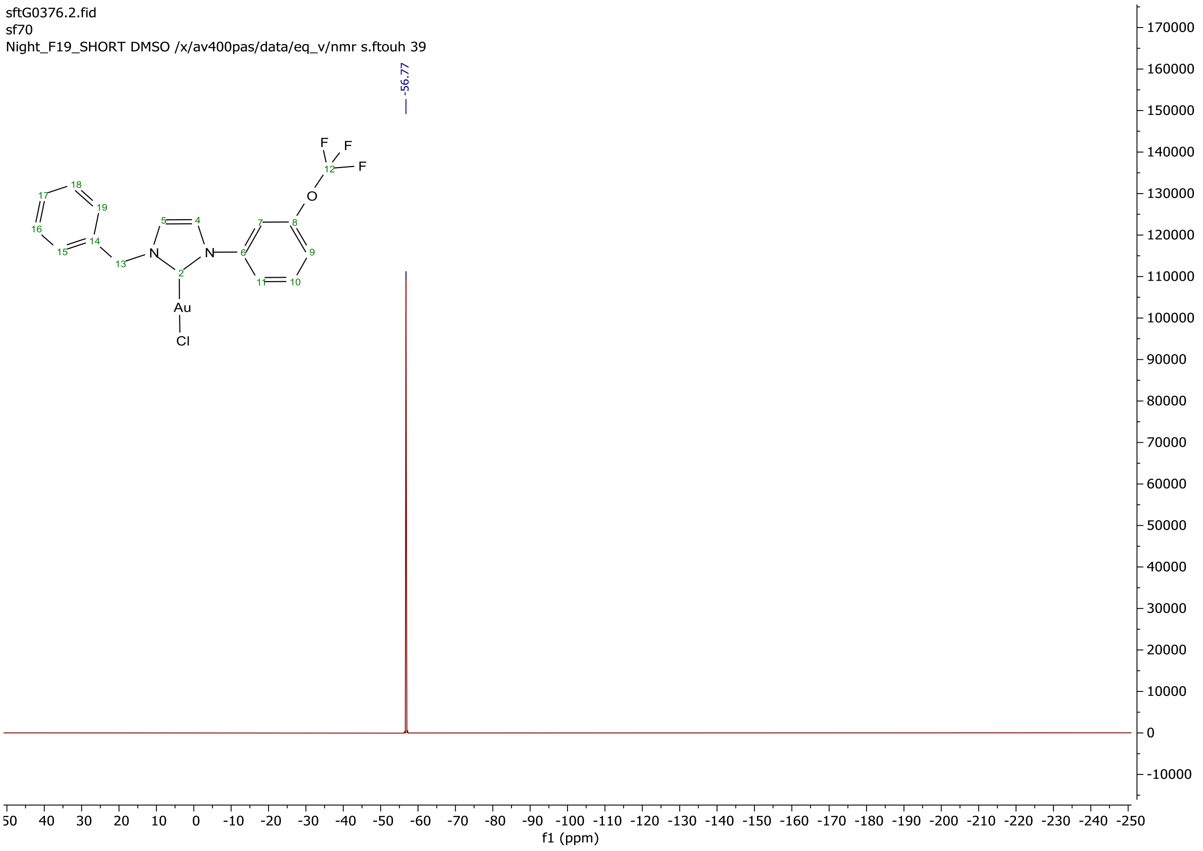

Figure S36: ^{19}F NMR spectrum of **16**.

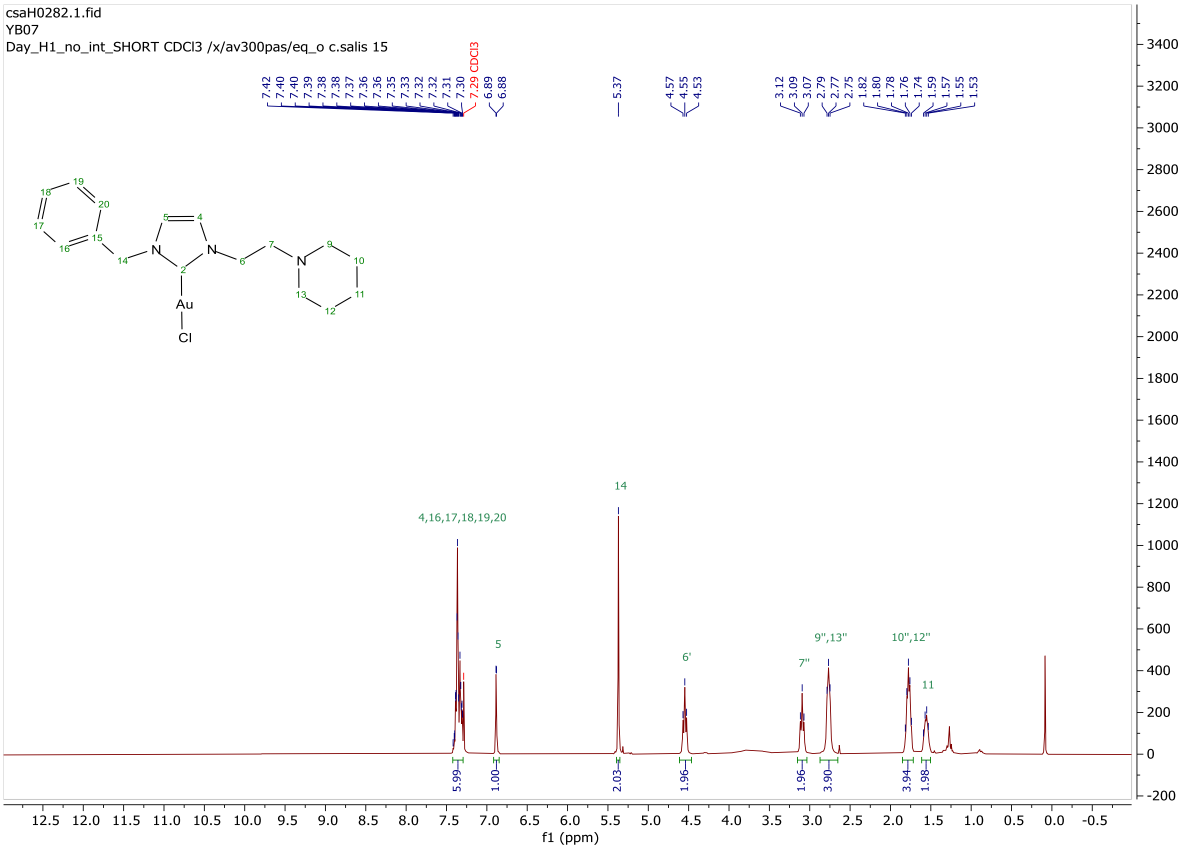

Figure S37: ^{1}H NMR spectrum of **17**.

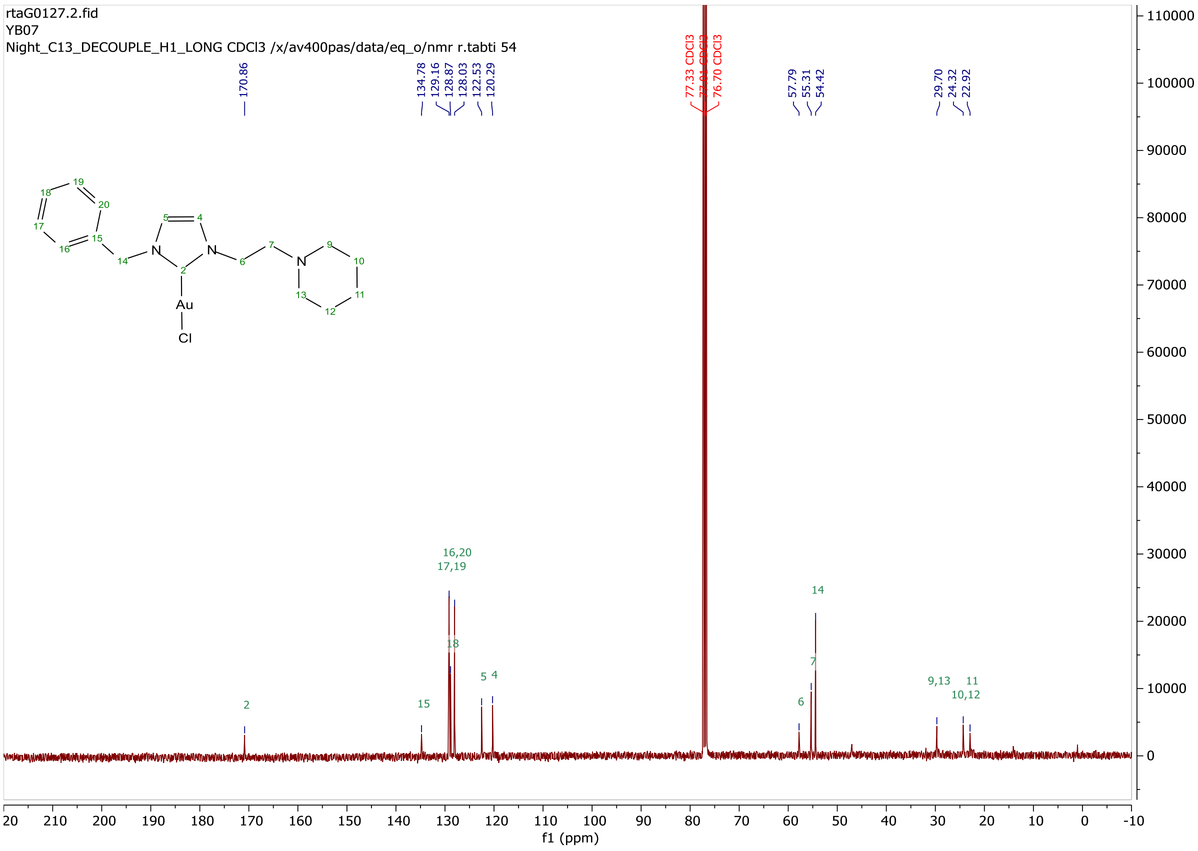

Figure S38: ^{1}H NMR spectrum of **18**.

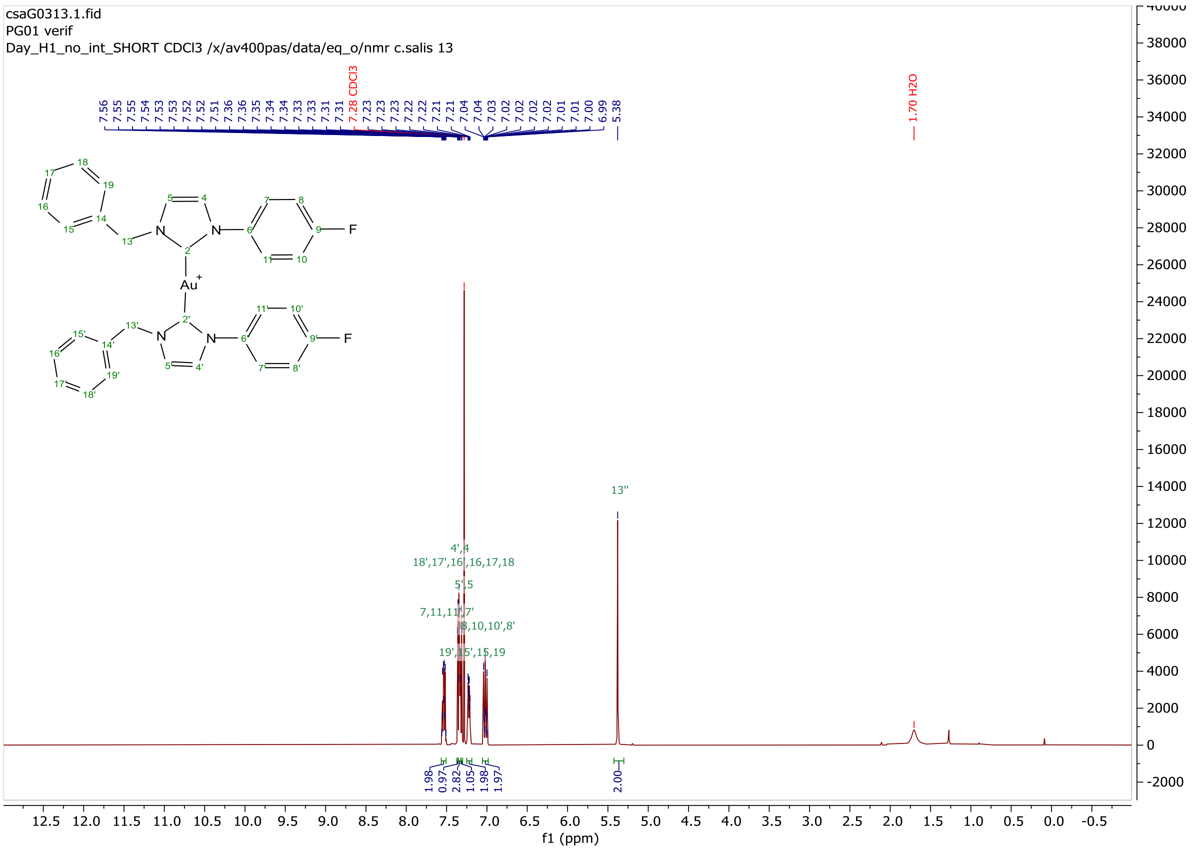

Figure S39: ^{1}H NMR spectrum of **19**.

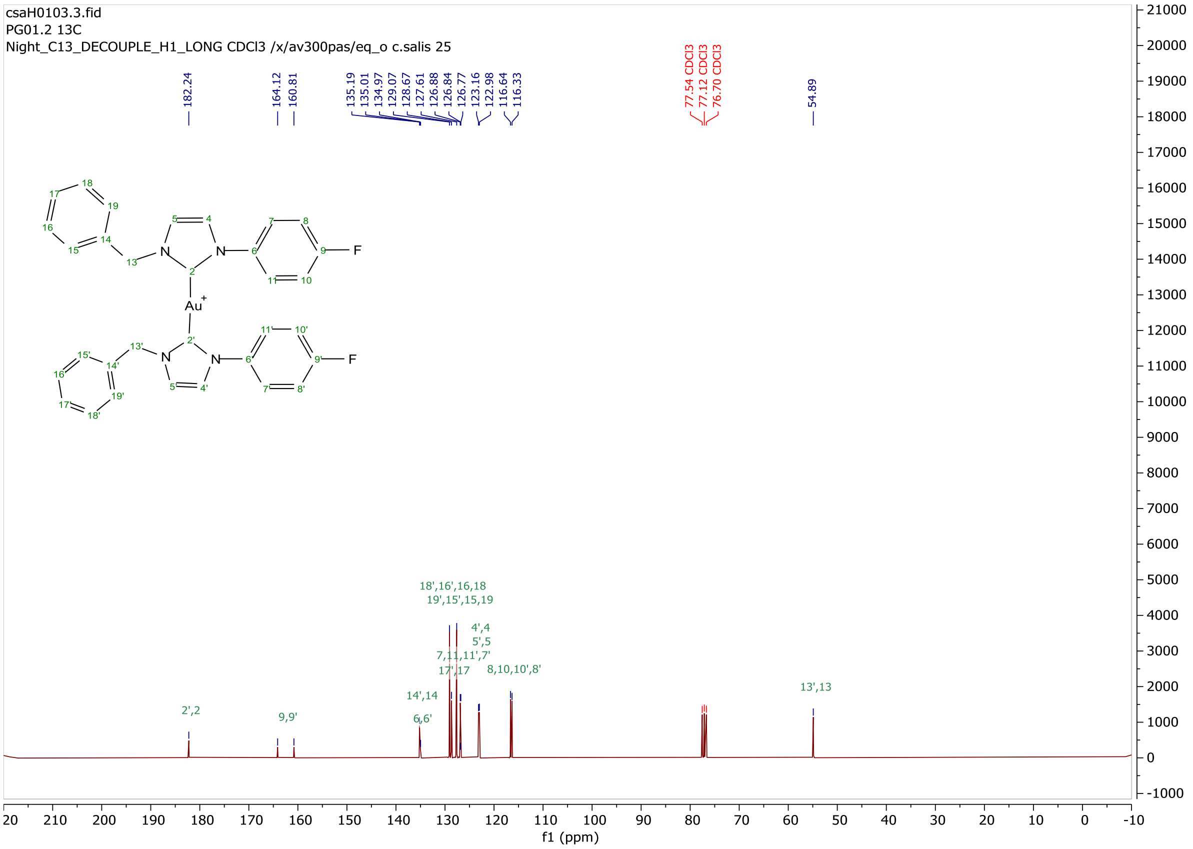

Figure S40: ^{13}C NMR spectrum of **19**.

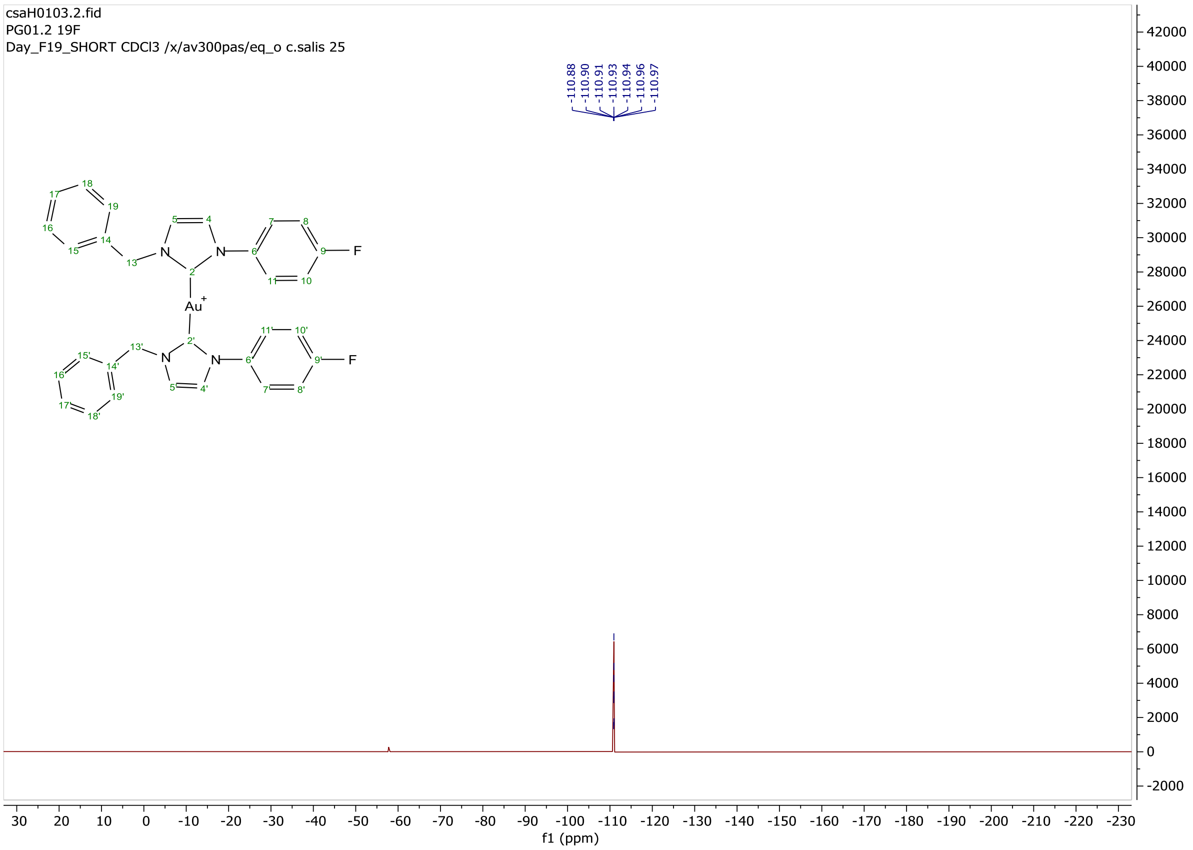

Figure S41: ^{19}F NMR spectrum of **19**.
